# Virus-induced APOBEC3 transmutagenesis in bladder cancer initiation

**DOI:** 10.1126/sciadv.aea6124

**Published:** 2025-12-03

**Authors:** George H. Hatton, Sally R. James, Andrew S. Mason, Richard T. Gawne, Helena Vogel, Karen Hogg, Parisa Boukani, Gemma Swinscoe, Anjum Khan, Matthew Welberry Smith, Michael A. Carpenter, Omar Masood, Iñigo Martincorena, Andrew Macdonald, Reuben S. Harris, Gabriel J. Starrett, Jennifer Southgate, Simon C. Baker

**Affiliations:** ^1^ Jack Birch Unit for Molecular Carcinogenesis, Department of Biology and York Biomedical Research Institute, University of York, Heslington, York, UK.; ^2^ Bioscience Technology Facility, Department of Biology, University of York, Heslington, York YO10 5DD, UK.; ^3^ Laboratory of Cellular Oncology, Center for Cancer Research, National Cancer Institute, National Institutes of Health, Bethesda, MD, USA.; ^4^ Faculty of Biological Sciences, School of Molecular and Cellular Pathology, University of Leeds, Leeds, UK.; ^5^ Department of Haematology, St. James’s University Hospital, Leeds, UK.; ^6^ Leeds Institute of Medical Research, University of Leeds, Leeds, UK.; ^7^ Department of Biology and York Biomedical Research Institute, University of York, Heslington, York, UK.; ^8^ Leeds Kidney Unit, St. James’s University Hospital, Leeds Teaching Hospitals NHS Trust, Leeds, UK.; ^9^ Department of Biochemistry and Structural Biology, The University of Texas at San Antonio, University of Texas, San Antonio, TX, USA.; ^10^ Howard Hughes Medical Institute, The University of Texas at San Antonio, San Antonio, TX, USA.; ^11^ Cancer, Ageing and Somatic Mutation, Wellcome Sanger Institute, Hinxton, UK.

## Abstract

Carcinogenesis in human urothelium is driven by a high burden of mutations caused by the antiviral “APOBEC3” (apolipoprotein B mRNA editing enzyme, catalytic subunit–like 3) cytosine deaminase enzymes; however, there is no established viral etiology. BK polyomavirus (BKPyV) is a ubiquitous childhood infection that persists in the kidney during adulthood and is frequently detected in urine. Chronic BKPyV infections of normal human urothelium induced an innate response, including apical extrusion of infected cells. Local paracrine interferon signaling induced APOBEC3 expression in both infected and juxtaposed bystander cells, leading to acquisition of hallmark APOBEC3-mediated mutational signatures that recapitulated the variation in mutational character found in patients with muscle-invasive bladder cancer. In our model for urothelial carcinogenesis, uninfected bystander cells witnessing BKPyV infection become APOBEC3 damaged, escape extrusion, and acquire hypermutable advantage. “Transmutagenesis” explains how cells proximal to infected neighbors acquire cancer-initiating mutations. This hypothesis for how urothelial cancers can develop as APOBEC3 signature rich, while remaining virus-negative, suggests that a large proportion of urothelial carcinomas may be preventable by antiviral intervention.

## Teaser

A model for hit-and-run viral carcinogenesis in the bladder.

## INTRODUCTION

Humans have coevolved with common viral infections that can persist in specific tissues with unknown consequences. During periods of immune limitation (such as coinfection or immunosenescence) or as a consequence of therapeutic immunosuppression associated with organ transplantation, persistent viral infections can reemerge to cause pathologies. BK polyomavirus (BKPyV) is a common childhood infection that reaches a seroprevalence of 95% in UK adults (*1*) and persists asymptomatically in the renal tubular epithelium (*2*). BKPyV DNA can be detected in the urine of the general population at a frequency that increases with age (*3*). Under immunosuppression for organ transplantation, the reactivation of persistent BKPyV infections is common and associated with an increased risk of nephropathy (*4*–*6*), ureteric strictures (*7*, *8*), hemorrhagic cystitis (*9*), and bladder cancers (*10*, *11*), where viral DNA is sometimes integrated into the tumor genome (*12*).

The apolipoprotein B mRNA editing enzyme, catalytic subunit–like (“APOBEC”) family of single-stranded DNA cytosine deaminases are innate immune effectors that act to damage viral genomes, preferentially targeting 5′-TCW-3′ motifs (where W = A/T) in single-stranded DNA and transforming cytosine into uracil. The role of APOBEC3 enzymes in limiting viral replication was first identified for HIV (*13*) but has since been extended to a diverse range of particularly, but not exclusively, DNA-based viruses [reviewed (*14*)]. The induction of APOBEC3 enzymes as a host response to infection is thought to have played an important role in limiting Polyomaviridae infections in evolutionary history, reducing TCW motifs in viral genomes (*15*, *16*). The question of whether virally induced APOBEC3s damage the host human genome has been inferred from forced overexpression studies (*17*, *18*), but the capacity for endogenous activity to cause mutational damage has yet to be confirmed. APOBEC3 signature TCW mutations characterize carcinogenesis in human urothelium (*19*–*21*); however, no APOBEC3-inducing mechanism has been established to drive initiation. Human models of urothelial carcinogenesis are necessary because BKPyV only infects human cells, and animal models lack closely related orthologs of the APOBEC3A and APOBEC3B enzymes that are up-regulated by human cells in response to BKPyV infection (*22*). The present study fills the mechanistic gap between BKPyV and bladder cancers (where the vast majority are virus negative) by demonstrating that the APOBEC3 mutational signatures observed in tumors can be recapitulated in uninfected bystander urothelial cells responding to a juxtaposed infection.

## RESULTS

### Innate antiviral signaling drives APOBEC3 expression

**Fig. 1.**
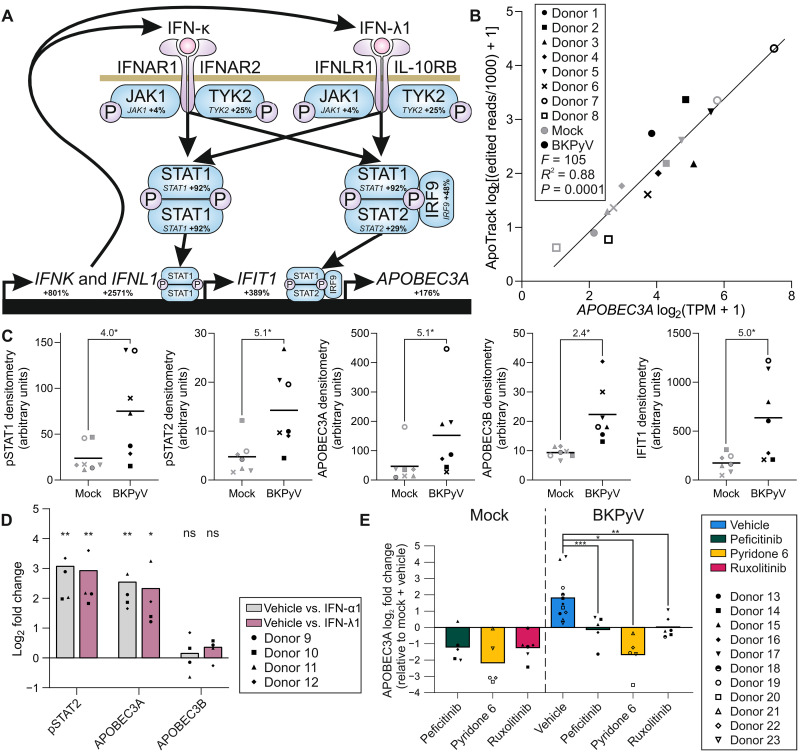
Innate antiviral signaling during BKPyV infection of normal human urothelium. (**A**) mRNA-seq of dNHU cell cultures at 21 dpi with BKPyV (Dunlop strain) showed increases in transcript expression of components of the proposed pathway shown as percentages compared to mock-infected cultures (*n* = 8 independent NHU cell lines). IFNAR1, interferon-alpha/beta receptor 1; IFNAR2, interferon-alpha/beta receptor 2; IFNLR1, interferon-lambda receptor 1; IL-10RB, interleukin-10RB; IRF9, interferon regulatory factor 9; TYK2, tyrosine kinase 2. (**B**) Editing of mRNA by APOBEC3A was assessed using ApoTrack and was significantly predicted by *APOBEC3A* TPM in a simple linear regression analysis [*n* = 16 samples of mock- and BKPyV-infected dNHU cell cultures at 21 dpi from eight independent NHU cell lines, individually identified using a data point style that also applies to (C)]. (**C**) Western blotting of matched lysates from the same donors as the mRNA-seq study showed significant increases in the phosphorylation of signal transducer and activator of transcription 2 (STAT2) and expression of APOBEC3A, APOBEC3B, and IFIT1 at 21 dpi with BKPyV (*n* = 7 independent NHU cell lines). The numbers above the graphs indicate fold change (blots are provided as fig. S2). (**D**) Lysates from dNHU cell cultures treated with exogenous interferon-α1 (IFN-α1) (type I) or IFN-λ1 (type III) revealed increased phosphorylation of STAT2 and expression of APOBEC3A but not APOBEC3B (*n* = 4 independent NHU cell lines; blots are provided as fig. S2). (**E**) Inhibition of APOBEC3A via Janus kinase (JAK) inhibition (by 2.5 μM peficitinib, 2.5 μM pyridone 6, or 1 μM ruxolitinib) to block the interferon signaling cascade (*n* ≥ 5 independent NHU cell lines). JAK inhibition also led to significantly decreased phosphorylation of STAT2 and reduced expression of IFIT1; however, no change was observed in APOBEC3B expression (fig. S3). Statistical significance is represented as follows: *<0.05, **<0.01, ***<0.001. ns, not significant.

messenger RNA sequencing (mRNA-seq) of mitotically quiescent and functionally differentiated normal human urothelial (dNHU) cell cultures infected with the BKPyV analyzed at 21 days postinfection (dpi) highlighted increased interferon signaling (Fig. 1A and fig. S1), notably less apparent in more acute studies (*22*) or other models (*23*, *24*). Increased expression of *IFNK* and *IFNL1* interferon transcripts during infection suggested that combined type I and type III interferon signaling mediates the innate response (exemplified by the interferon-induced protein with tetratricopeptide repeats 1 gene *IFIT1*, a canonical interferon-stimulated gene) and *APOBEC3A* transcript expression (Fig. 1A and fig. S1). APOBEC3A activity was confirmed using ApoTrack (*25*), which found a 2.4-fold increase in deaminated cytosines in RNA hairpins (*P* = 0.014; fig. S1), and this activity was significantly predicted by *APOBEC3A* transcripts per million (TPM; Fig. 1B).

Immunoblotting confirmed that BKPyV infection of dNHU cell cultures stimulated significant increases in the phosphorylated forms of signal transducer and activator of transcription 1 and 2 (pSTAT1 and pSTAT2) and expression of APOBEC3A, APOBEC3B, and interferon-induced protein with tetratricopeptide repeats 1 (IFIT1) proteins (Fig. 1C and fig. S2). pSTAT1 and pSTAT2 were predictive of APOBEC3A expression, and IFIT1 protein abundance correlated with that of APOBEC3A (fig. S2). Exogenous application of interferon-α1 (IFNα1) (type I) or IFN-λ1 (type III) to uninfected dNHU cell cultures led to significantly increased pSTAT2 and expression of APOBEC3A but not APOBEC3B (Fig. 1D and fig. S2). Furthermore, competitive Janus kinase (JAK) inhibitors (peficitinib, pyridone 6, and ruxolitinib) prevented increases in BKPyV-induced pSTAT2 and the induction of APOBEC3A and IFIT1 but had no effect on BKPyV-induced APOBEC3B expression (Fig. 1E and fig. S3).

### Infection-induced APOBEC3 mutational signatures

**Fig. 2.**
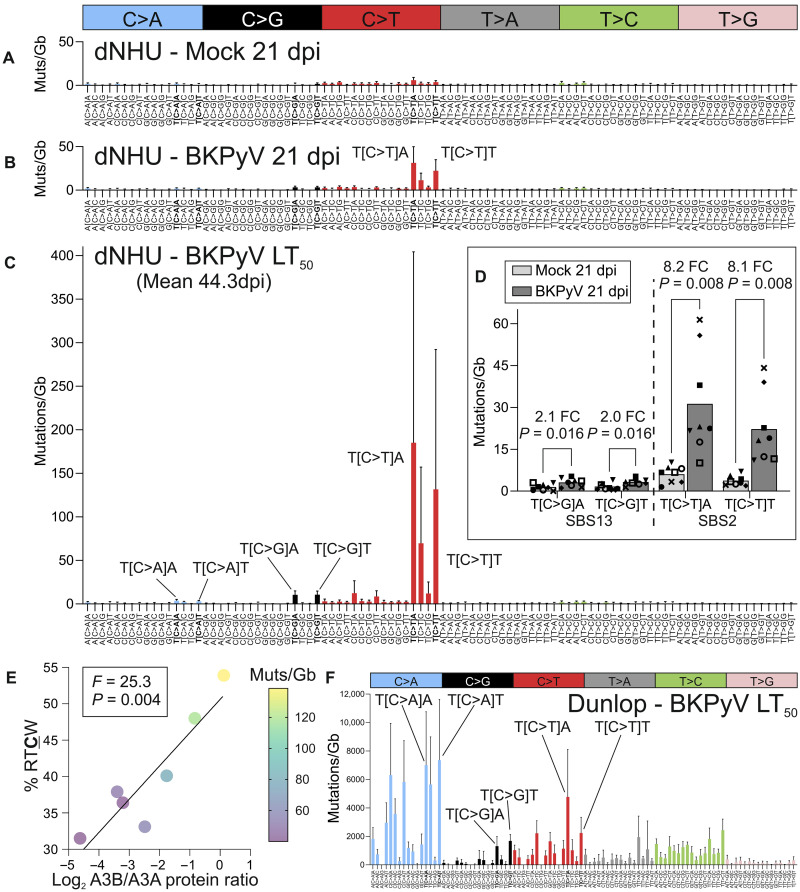
Mutational signature analysis reveals that dNHU cell genomes are damaged by APOBEC3 enzymes during BKPyV infections. NanoSeq of mock and BKPyV [Dunlop; multiplicity of infection (MOI) = 0.01]–infected dNHU cell cultures was used to derive mutational signatures (**A** to **C**) at 21 dpi and a time point where 50% of the culture had been lost to lytic infection (LT_50_). The plot labels highlight the APOBEC3A/B TCW mutations (Muts) (where W = A/T) used in later analyses. Plots showing the individual donors with distinct point identifiers are provided as fig. S4. (**D**) Fold changes and significance testing (Wilcoxon) for mock- and BKPyV-infected cultures at 21 dpi. Bar denotes the mean, and each independent donor (*n* = 8) has a distinct point identifier. FC, fold change. (**E**) Scatter plot showing the log_2_-transformed Western blot–derived ratio of APOBEC3A (A3A) to APOBEC3B (A3B) proteins against the percentage of purines in the −2 position (RTCW APOBEC3B-characteristic mutations; where R = A/G). The plot suggests that donors expressing a higher amount of APOBEC3B have both a higher % RTCW and a higher overall mutational burden (shown as a heatmap of the point color). (**F**) Mutational signature in the BKPyV Dunlop genome during dNHU cell culture infections at the LT_50_ showed a limited contribution from APOBEC3 activity (*n* = 7).

dNHU cell cultures were infected with BKPyV and maintained to 21 dpi or until 50% of the culture had been lost to lytic infection “LT_50_” (Fig. 2, A to C). Nanorate sequencing (NanoSeq) (*26*)–derived mutational signatures were compared against the Catalogue of Somatic Mutations in Cancer (COSMIC) single-base substitution (SBS) database. At 21 dpi, SBS2 mutations (T[C>T]A and T[C>T]T) and SBS13 mutations (T[C>G]A and T[C>G]T) were significantly increased by 8.2-, 8.1-, 2.1-, and 2.0-fold, respectively (Fig. 2D). SBS2 mutations form through a combination of normal base pairing of the APOBEC3-generated uracil (which is an “instructional” lesion) with adenine during DNA replication and A-rule repair opposite abasic sites following uracil excision. By contrast, SBS13 derives from repair of abasic sites by error prone polymerases (*27*). The greater induction of SBS2 over SBS13 requires further investigation but suggests excision of uracils before DNA replication was limited (Fig. 2, B and C).

Further evidence of APOBEC3 damage to the genome comes from the suggestion of double-base substitution (DBS) signature of CC>TT and TG>CA mutations similar to COSMIC “DBS11,” which has previously been associated with APOBEC3A/B mutagenesis in cancers (*19*), including nearly half the bladder cancer samples tested in one study (*28*); however, the induction of CC>TT changes was not statistically significant at 21 dpi due to the low mutation count (fig. S5). An insertion/deletion (ID) signature of single cytosine deletions also appeared to be developing (fig. S5), similar to COSMIC “ID9” and as described prospectively for APOBEC3A overexpression (*29*).

The extended nucleotide context around APOBEC3 signature mutations, and particularly the percentage of purines (R = A/G) at the −2 position (“RTCW”), is instructive of which enzyme is dominating the process. In this mixed endogenous APOBEC3A/APOBEC3B expression model (*18*), the mean RTCW percentage for all eight donors at 21 dpi was 39.4% (fig. S6). Data from Hap1 cells suggest that overexpressing APOBEC3A gives ~30% RTCW, while overexpressing APOBEC3B led to ~50% RTCW (*18*). To further investigate the interdonor heterogeneity present in the dNHU cell cultures, we plotted the RTCW percentage against the Western blot–derived ratio of APOBEC3B/APOBEC3A protein, which supported the concept that most donors showed mixed mutagenesis, with a slight dominance by APOBEC3A (Fig. 2E). However, ApoTrack estimated that APOBEC3A mRNA editing was not significantly predictive of RTCW DNA mutations/gigabase or RTCW percentage, suggesting that RNA deamination and DNA mutation rates are not proportional (fig. S6).

Two donors (donors 4 and 6) showed higher APOBEC3B protein abundance, the ~50% RTCW characteristic of APOBEC3B (mean = 51.0%) and the highest mutational burdens of all donors analyzed (Fig. 2E and fig. S6). This finding contrasts with overexpression data, suggesting that APOBEC3A is more mutagenic (*18*), potentially because of the presence of APOBEC3B in infected cells where viral large T antigen (LT-Ag) manipulates both deaminase expression (*30*) and DNA repair processes.

While there is evolutionary evidence for the mutagenesis of polyomavirus genomes by APOBEC3 enzymes (*15*, *16*), knockdown studies suggest that the APOBEC3s exert limited effect on BKPyV infection (*16*). We observed APOBEC activity against the BKPyV genome as a minor component of a mutational signature dominated by C>A transversions in the dNHU cell cultures (Fig. 2F). The BKPyV Dunlop genome mutational signature suggests a combination of SBS18 and SBS36 that are thought to be derived from reactive oxygen species–mediated formation of 8-oxoguanine, such as that triggered by hydrogen peroxide (*31*).

### *APOBEC3A/B* knockout ablates signature formation

**Fig. 3.**
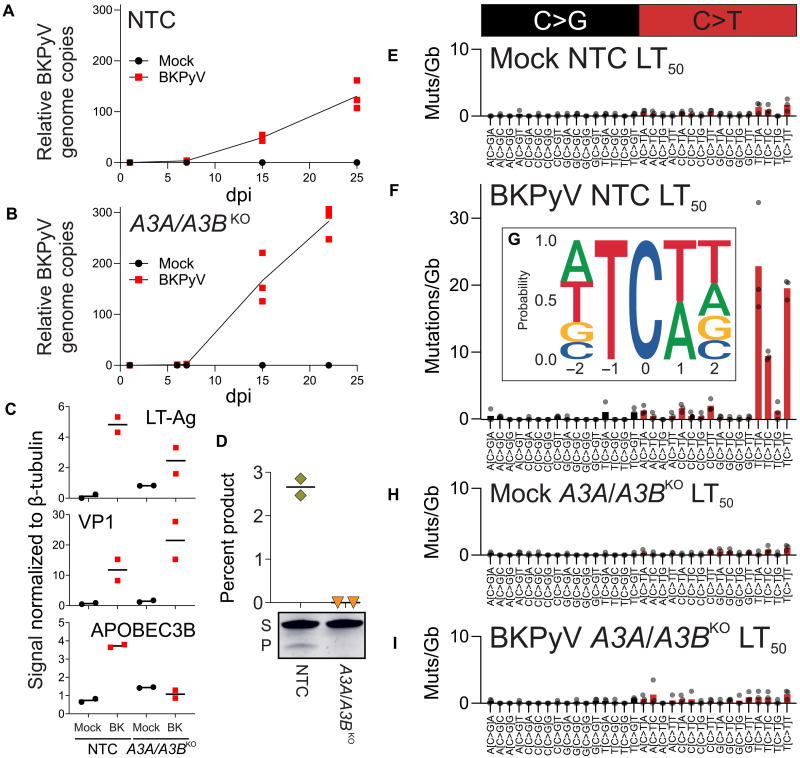
Analysis of HBLAK model of CRISPR-mediated APOBEC3A and APOBEC3B knockout. Clonal HBLAK cultures previously CRISPR-edited with either (**A**) nontargeted control (NTC) or (**B**) *APOBEC3A* and *APOBEC3B* gene knockout (*A3A*/*A3B*^KO^) guide RNAs (gRNAs) were mock or BKPyV (Gardner strain; MOI = 0.5) infected, and viral genome copies were assessed by quantitative polymerase chain reaction (qPCR) (*n* = 3). (**C**) Western blotting densitometry confirmed viral infection by detection of LT-Ag and viral capsid major protein 1 (VP1) proteins. APOBEC3B was induced by nontargeted control cells in response to BKPyV infection but not in the *A3A/A3B*^KO^. APOBEC3A was not detectable in HBLAK cell lysates (*n* = 2; exemplar Western blot shown in fig. S8). (**D**) Deaminase activity assays confirmed the knockout of APOBEC3 function in PMA-treated *A3A/A3B*^KO^ HBLAK cells, with the exemplar gel showing bands for substrate (S) in both sample types and deamination-dependent product (P) only in NTC lysates (*n* = 2). Mutational signatures were derived by NanoSeq at the time point, where 50% of the culture had been lost to lytic infection LT_50_, and are expressed as the count for each mutation type divided by the number of gigabases from the NanoSeq library that was sequenced. *n* = 3 for each panel, and bars denote the mean. Mutational signatures for (**E**) mock infection of nontargeted control cells and (**F**) BKPyV infection of nontargeted control cells. (**G**) Five-base footprint for APOBEC signature TCW context mutations in BKPyV infection of nontargeted control cells. Mutational signatures for (**H**) mock infection of *A3A/A3B*^KO^ cells and (**I**) BKPyV infection of *A3A/A3B*^KO^ cells.

Infection of the immortalized HBLAK human urothelial cell line (*32*) was used as a genomically tractable model for the development of simultaneous CRISPR-mediated APOBEC3A and APOBEC3B gene knockouts (*A3A/A3B*^KO^) (fig. S7). Clones derived from the HBLAK line were infected with BKPyV [Gardner strain; multiplicity of infection (MOI) = 0.5] and infections progressed with increasing viral genome copies to LT_50_ by 25 dpi (Fig. 3, A and B). Induction of APOBEC3B (but not APOBEC3A) protein by HBLAK cells was observed in response to BKPyV infection in the nontargeted control clones, and this induction was not observed in the *A3A/A3B*^KO^ (Fig. 3C). Deaminase activity assays could not detect cytosine deamination in *A3A/A3B*^KO^ HBLAK cells treated with phorbol 12-myristate 13-acetate (PMA) as a positive control to induce robust APOBEC3B expression (Fig. 3D). NanoSeq revealed that APOBEC3A/B TCW mutations were significantly increased 5.9-fold in BKPyV-infected nontargeted control clones compared with mock-infected cultures (*P* = 0.014; Fig. 3, E and F, and fig. S8). The mean prevalence of RTCW mutations was 51.4% (Fig. 3G), characteristic of APOBEC3B-dominated systems (*18*). *A3A/A3B*^KO^ abolished the induction of TCW mutations when comparing mock- to BKPyV-infected HBLAK cultures (*P* = 0.362; Fig. 3, H and I, and fig. S8), demonstrating that APOBEC3A/B function was critical to the formation of SBS2 signature mutations in human urothelial cells. During HBLAK infections, the Gardner BKPyV genome accumulated mutations with the same pattern of C>A transversions observed in the dNHU cell model of Dunlop BKPyV infection; however, there was no evidence of APOBEC3-derived C>G or C>T mutations in the Gardner genome (fig. S8).

### APOBEC3 expression in the virus extrusion microenvironment

**Fig. 4.**
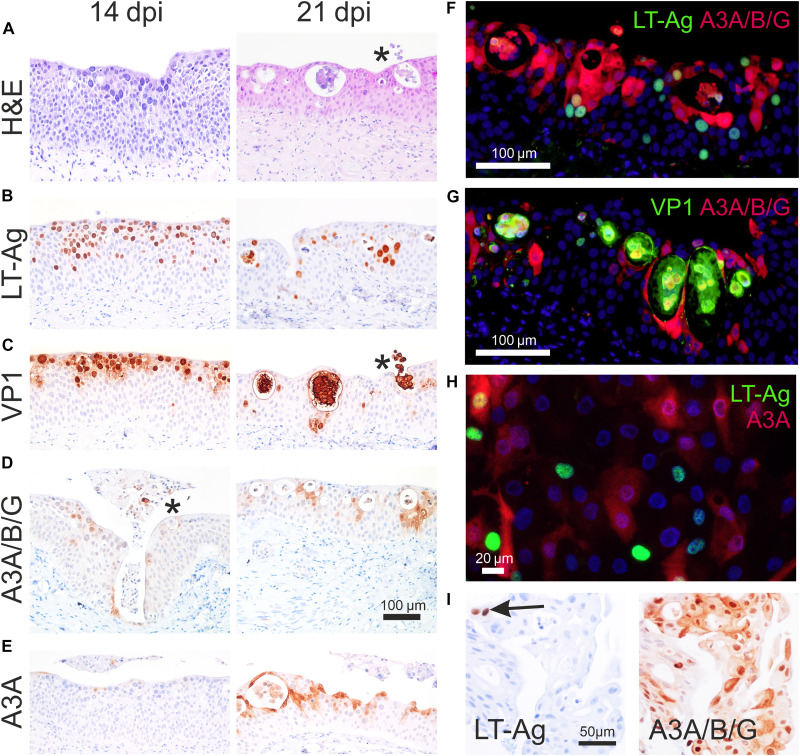
Immunolabeling of NHU cells infected with BKPyV. (**A** to **G**) Normal human ureteric organ culture infected apically with BKPyV (Dunlop; *n* = 3 independent donors). (A) Hematoxylin and eosin (H&E) staining highlights the larger, darker nuclei of infected cells that have been through S phase. Asterisk denotes regions with apical extrusion of infected cells. (B) LT-Ag and (C) VP1 showed widespread nuclear labeling at 14 and 21 dpi. VP1 highlighted the isolation and apical extrusion of late-stage infection cells by the urothelium. (D) Immunoperoxidase labeling of a shared epitope in APOBEC3A/B/G (A3A/B/G) enzymes (*37*) and (E) labeling of APOBEC3A (A3A) with a monospecific antibody (*18*) showed no detectable labeling at 14 dpi except around a single area of apical extrusion (asterisk). By 21 dpi, APOBEC3-labeling was widespread surrounding areas of isolation and extrusion of infected cells. Scale bar in (D) applies to all images in (A) to (E). Colabeling of APOBEC3A/B/G enzymes with (F) viral LT-Ag and (G) VP1 showed limited instances of colocalization to the same cell. APOBEC3A/B/G expression was most commonly found in uninfected urothelial cells involved in the processes of isolation and apical extrusion of late-stage infection cells. (**H**) Colabeling of APOBEC3A and LT-Ag in dNHU cell cultures at 21 dpi showed limited instances of colocalization to the same cell (*n* = 3 independent donors). (**I**) Serial sections of BKPyV-associated hemorrhagic cystitis showing two cells positive for LT-Ag (arrowed) and APOBEC3A/B/G labeling that extends into virus-negative bystander urothelial cells (fig. S11).

A primary normal human ureter organotypic culture model of BKPyV infection was developed (hereafter “organ cultures”). Organ cultures recapitulate key features of clinical BKPyV infections (*33*), including nuclear inclusions and/or anisokaryosis (Fig. 4A and fig. S9). In organ culture, BKPyV infection progressed over a similar time course to the dNHU cell cultures, with signs of lytic infection apparent at 21 dpi (Fig. 4, A to C). Immunohistologically, canonical features of Polyomaviridae infection including phosphorylation of retinoblastoma protein, enhancer of zeste 2 polycomb repressive complex 2 subunit (EZH2) accumulation, and Ki67 positivity (displaying the large nuclear granules characteristic of the G_2_ cell cycle phase) were observed in organ cultures (fig. S10), as previously described in dNHU cell cultures (*22*). Apical extrusion of cells displaying signs of late stage infection (frequently exhibiting karyolysis) was observed in some organ cultures (Fig. 4, A, C, and D, asterisk). Apical extrusion is a feature of clinical BKPyV infection, which generates “decoy cells” detected by urine cytology from renal proximal tubular epithelial (RPTE) cells (*34*) and is conserved across other epithelium/virus combinations (*35*, *36*). In both the organ cultures and dNHU cell cultures, APOBEC3 enzymes were not induced by initial exposure to BKPyV virions or the precytopathic phase of infections as denoted by expression of LT-Ag or viral capsid major protein 1 (VP1) proteins (Fig. 4, B to E, and fig. S9). In the organ cultures, the local microenvironment of infected cell isolation and apical extrusion was surrounded by cells displaying increased, and highly localized, expression of APOBEC3 proteins (Fig. 4D). Additional labeling of organ cultures with an APOBEC3A-specific antibody (*18*) showed a similar localization pattern to the APOBEC3A/B/G-shared epitope antibody (*37*) (Fig. 4E). The pattern of APOBEC3 induction in dispersed foci may help explain why clonal APOBEC3 signature–positive patches in the normal urothelium were spatially separated (*38*). Direct examination of nuclei containing viral inclusions found them to have immunolabeled with the APOBEC3A/B/G antibody while frequently negative with the APOBEC3A-specific antibody (fig. S9). Nuclear inclusions disperse APOBECs due to the densely tessellated virions, which suggests APOBEC3B induction in cells with late-stage viral infection (*22*, *30*).

In organ cultures, APOBEC3A/B/G labeling was frequently, but not exclusively, observed in “bystander” cells surrounding sites of active VP1^+^ cell isolation/extrusion, and the bystanders themselves were negative for viral proteins (LT-Ag or VP1; Fig. 4, F and G). The APOBEC3A^+^ bystander cells induced by 21 dpi in dNHU cell cultures were mostly LT-Ag negative (Fig. 4H). We hypothesize that the native tissue architecture of organ cultures reduces paracrine interferon diffusion leading to localized APOBEC3 induction (Fig. 4, D and E), whereas in dNHU cell cultures, the interferons are more freely diffusible and can induce APOBEC3 expression in more distant bystanders (Fig. 4H).

Clinical BKPyV infection of normal human urothelium has only rarely been described histologically, including the original discovery of the virus in the ureter of a kidney transplant recipient in 1971 (*33*), and has not been followed up because of a scarcity of relevant clinical biopsies. Patient biopsies containing active urothelial BKPyV infection are rare because their asymptomatic nature does not motivate invasive clinical investigation. In bone marrow transplant recipients, immunosuppression can lead to symptomatic BKPyV-driven hemorrhagic cystitis. Immunohistology of a BKPyV hemorrhagic cystitis bladder biopsy confirmed the presence of APOBEC3-positive urothelial cells that were negative for viral proteins (Fig. 4I, with further labeling of serial sections in fig. S11).

### Fluorescence-activated cell sorting–NanoSeq detection of mutations in bystander cells

**Fig. 5.**
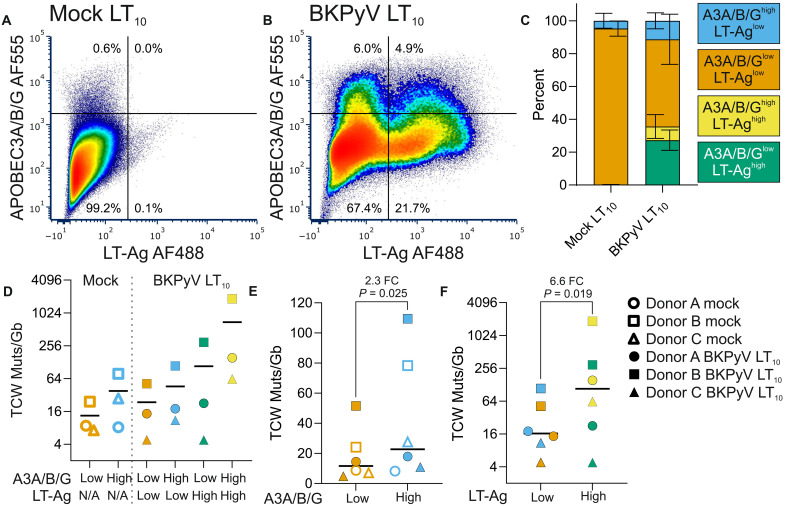
FACS-NanoSeq detection of mutations in bystander cells. (**A**) FACS dot plot of mock-infected dNHU cell cultures (gated for single cells) at an infection-matched LT_10_ (where 10% of the infected culture has been lost to cytopathic effects) reveals the presence of an APOBEC3A/B/G^high^ subpopulation. Cells were dual labelled for a shared epitope in APOBEC3A/B/G (A3A/B/G) with Alexa Fluor 555 (AF555) and viral Large T Antigen (LT-Ag) with Alexa Fluor 488 (AF488). (**B**) BKPyV Dunlop (MOI = 0.01) infection increased the APOBEC3A/B/G^high^/LT-Ag^low^ subpopulation at the LT_10_ and created LT-Ag^high^ populations. (**C**) The mean increase in the APOBEC3A/B/G^high^/LT-Ag^low^ bystander population during infection was 4.9-fold. The color legend in (C) applies to (C) to (F). (**D**) NanoSeq of FACS subpopulations at the LT_10_ showing that increased (**E**) APOBEC3A/B/G and (**F**) LT-Ag detection were both significantly associated with higher rates of mutation. The shapes legend in (F) applies to panels (D) to (F). FACS was performed in tissues from three independent NHU cell lines with full mutational signatures provided as fig. S12. N/A, not applicable.

To evaluate which populations were exposed to the risk of APOBEC3A/B-mediated mutations during BKPyV infections, we performed FACS at LT_10_ (when 10% of the culture had succumbed to lytic infection) on the basis of APOBEC3A/B/G and LT-Ag labeling intensity (Fig. 5, A and B, and fig. S12). The bystander (APOBEC3A/B/G^high^ and LT-Ag^low^) population was increased by BKPyV infection in all three donors (Fig. 5C, mean = 4.9-fold, and fig. S12) and APOBEC3A/B/G^high^ populations acquired 2.3-fold more TCW mutations than APOBEC3A/B/G^low^ (*P* = 0.025, paired *t* test; Fig. 5, D and E). There was no significant change in the rate of mutation in APOBEC3A/B/G^high^ and LT-Ag^low^ populations when comparing mock- and BKPyV-infected cultures (Fig. 5D); however, the increased numbers of uninfected APOBEC3A/B/G^high^ bystander cells in the urothelium during infection represent a relevant cancer initiation risk. Detection of LT-Ag labeling in cells was associated with a significant 6.6-fold increase in TCW mutations (*P* = 0.019 paired *t* test; Fig. 5F). The APOBEC3A/B/G^high^ and LT-Ag^high^ subpopulation gives further support to the emergence of the CC>TT-dominated DBS11 during BKPyV infections (fig. S12), with potential relevance to the CC242-243TT *TERT* mutations observed in bladder cancers (*39*).

### Is APOBEC3A or APOBEC3B more responsible for the mutations?

**Fig. 6.**
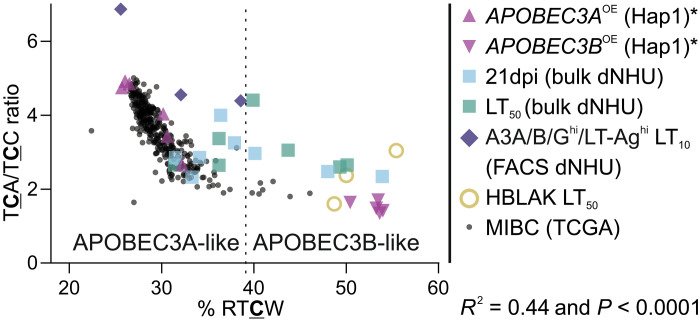
Mutagenesis in BKPyV infection models of human urothelium recapitulates that observed in bladder cancers. To assess the relative contribution of APOBEC3A and APOBEC3B to the mutational load of tissue models and cancer samples, we plotted the percentage of purines in the −2 position relative to TCW mutations “% RTCW” against the ratio of mutations within TCA/TCC trinucleotide contexts. These two differential properties of APOBEC3A- and APOBEC3B-generated mutations at a genome level separate Hap1 models specifically overexpressing each enzyme (purple) (*18*). Asterisk denotes that the Hap1 cell models generated C>A mutations not associated with APOBEC3 activity and they were therefore excluded from analysis to focus on C>G and C>T TCW mutations for this model only (*18*). BKPyV-infected HBLAK cultures (sand-colored open circles; Fig. 4), which only express APOBEC3B, cluster with the Hap1-APOBEC3B^OE^ model. MIBC samples from The Cancer Genome Atlas (TCGA) study (small gray; *n* = 357) (*21*) show a donor-specific profile with most APOBEC3A-dominated and some with more APOBEC3B-characteristic mutations. Bulk analysis of dNHU cell cultures in Fig. 3 at 21 dpi is shown in cyan, and the time point when 50% of the culture had been lost to lytic infection (LT_50_) is shown in teal. The APOBEC3A/B/G^high^ and LT-Ag^high^ FACS subpopulation from infected dNHU cell cultures in Fig. 5 is shown at the time point where 10% of the infected culture has been lost to cytopathic effects (LT_10_) and colored indigo. dNHU cell cultures from Fig. 3 (cyan/teal) and Fig. 5 (indigo) show a donor-specific diversity in APOBEC-characteristic mutations that covers the range seen in bladder cancer samples.

To determine whether APOBEC3A or APOBEC3B was the predominant deaminase, we compared our BKPyV infection models of urothelial carcinogenesis and patient bladder tumor data (*21*) to a Hap1 cell line model [with overexpression of *APOBEC3A* or *APOBEC3B* in isolation (*18*); Fig. 6]. The most robust separation of APOBEC3A (top left) from APOBEC3B-characteristic mutations (bottom right) in the Hap1 model was achieved using the ratio of TCA/TCC (*y* axis) and the percentage of purines in the −2 position relative to TCW mutations (“% RTCN”; Fig. 6). In the human genome (GRCh38), the ratio of TCA/TCC trinucleotides is 1.27, suggesting that APOBEC3B has a mild preference for mutating TCA, whereas it is the strong preference of APOBEC3A. The % RTCW in the human genome is 39.1% (marked as the dashed vertical line in Fig. 6), and, here, APOBEC3B shows preference for deaminating cytosines in a RTCW context, while APOBEC3A favors YTCW. The HBLAK urothelial model data (Fig. 3) clustered with the Hap1 *APOBEC3B*-overexpressing cells because HBLAKs only express APOBEC3B during BKPyV infection (Fig. 6). Overlaying the dNHU cell culture data from bulk populations (Fig. 2) and FACS APOBEC3A/B/G^high^ and LT-Ag^high^ subpopulations (Fig. 5) showed a broad spread that described the ranges seen in bladder cancer samples. In muscle-invasive bladder cancer (MIBC), TCW mutations were, on average, 36% of the tumor mutational burden (range = 8 to 74%). Neither the absolute numbers of TCW mutations nor the APOBEC3A/APOBEC3B character of MIBC samples were significantly different between the consensus molecular subtypes (fig. S13), suggesting that cancer phenotype is a unique and plastic interplay between mutations and niche, rather than deriving from different carcinogenic mechanisms. One MIBC sample had the BKPyV genome integrated into the host’s and had robust APOBEC3A-characteristic mutations (fig. S13). These data suggest that patient diversity in the innate response to BKPyV infection may drive the APOBEC3A response of the urothelium and influence the lifetime cancer risk for an individual.

## DISCUSSION

**Fig. 7.**
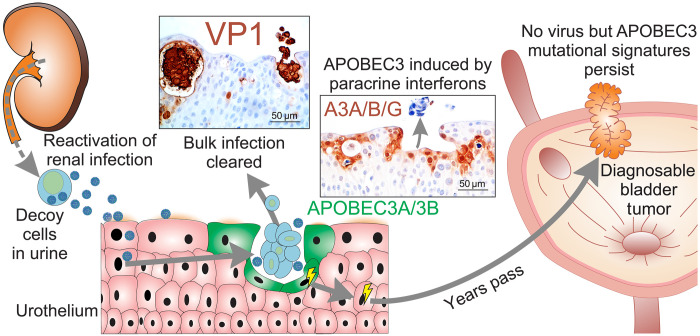
Model of APOBEC3-mediated bladder cancer initiation in the virus-infected cell extrusion microenvironment. Summary schematic of a model for bladder cancer initiation where lifelong, persistent BKPyV infections of the renal proximal tubular epithelium reactivate and shed decoy cells into the urine infecting the urothelium. As the infection progresses to lytic virion release, the urothelium isolates and apically extrudes infected cells (marked by expression of the VP1). In the viral extrusion microenvironment, bystander cells express interferon-regulated APOBEC3A, which damages the host genome. A period of episodic infections in which the cell mounts an APOBEC3A/B response leads to the accumulation of initiating DNA damage, which may persist as uracil lesions until the cell is required to divide. This hypothesis leads to a tumor defined by APOBEC3A/B mutagenesis but where no virus is present in the final cancer.

Urothelial carcinogenesis is driven by a high burden of mutations caused by antiviral APOBEC3-mediated cytosine deamination (*40*), but the disease has lacked a clear viral etiology due to the absence of viral RNA/DNA in tumors (*21*, *41*). On the basis of the findings reported here, we propose a model for urothelial carcinogenesis in the viral extrusion microenvironment created by BKPyV infections (Fig. 7). Persistent BKPyV infections of the renal proximal tubular epithelium are known to reactivate [in association with aging (*3*)], and infected decoy cells shed into the urine, infecting the urothelium. At the later cytopathic stages of infection, paracrine type I/III interferon signaling mediated local APOBEC3A/B mutagenesis in uninfected bystander cells “witnessing” infection in the viral extrusion microenvironment. The robust APOBEC3A/B response to BKPyV infections in bystander cells gives urothelial carcinogenesis a distinctive mechanism, with the mutations occurring in trans to the infection: “transmutagenesis.” Carcinogenesis by episomal BKPyV infections contrasts with tumors where the viral genome is integrated into the initiated cell and the APOBEC3A/B mutations (*12*, *42*–*44*) occur in cis (*45*) (hereafter “cismutagenesis”). Although we can only model a single-hit infection in vitro, the mitotically quiescent nature of the urothelium (*46*) means that the transmutagenesis burden likely accumulates unrepaired on the genome over a series of episodic BKPyV reactivations covering a period of many months until a “promotion” event where tissue repair necessitates cell cycle entry and DNA repair (*47*). We further hypothesize that the cell loss from the urothelium during lytic BKPyV infections may additionally serve as the promotion event required to stimulate proliferation in the urothelium and drive the initial outgrowth of clones with hypermutable competitive advantages. At present, because of a lack of effective BKPyV therapeutics, there is no way to cure an immunocompromised in vitro model of BKPyV infection (for colony-forming or xenotransplantation assays) and all cultures, or derived clones, end in a complete cytopathic death reminiscent of hemorrhagic cystitis. However, we hypothesize that the process outlined leads to a tumor defined by APOBEC3A/B mutagenesis but where no virus is present in the final cancer (Fig. 7). The interferon-mediated nature of this model suggests that these findings will have implications both for other viruses and infections of different tissues.

On the basis of cells from multiple human individuals, this study reveals how host variation influences outcome based on the mixed donor-specific burden of APOBEC3A and APOBEC3B mutations with the potential to initiate carcinogenesis. While normal human cells are known to be refractory to transformation in vitro, this study provides a mechanism for the APOBEC3A/B premalignant changes that underpin the creation of many bladder cancer driver genes, such as the most common protein-coding mutation in bladder cancer, which changes Fibroblast Growth Factor Receptor 3 serine 249 into a cysteine “*FGFR3*^S249C^” (*48*–*50*).

An alternative scenario for initiation could be that uninfected APOBEC3^high^ bystander cells subsequently become infected and a combination of high APOBEC3A/B cismutagenesis and LT-Ag–mediated tolerance of DNA damage leads to a “big bang” of mutations occurring in a single episode. BKPyV LT-Ag acts as an oncogene in the rare cases where it is permanently integrated into the host genome of bladder cancers (*12*); by contrast, in the BKPyV-driven cismutagenesis hypothesis, transient LT-Ag sequestration of p53 and inhibition of its transcription factor activity (*22*) may create an environment that supports carcinogenesis by allowing normal cells to survive the acquisition of the high mutational burdens associated with bladder tumors. In the DNA of cancer samples, the rapid accumulation of big bang cismutagenesis may be genomically indistinguishable from episodic bystander transmutagenesis lesions followed by bulk repair during tissue regeneration. Bladder cancers are largely negative for viruses (*20*), challenging the cismutagenesis hypothesis by requiring the initiated cell to both eliminate/abort the infection and avoid extrusion in the innate urothelial response.

A limitation of this kind of work is the assertion that bystander cells were truly uninfected. However, because APOBEC3A/B enzymes were not induced during initial infection of urothelial tissue or organ cultures by BKPyV, it is implausible that APOBEC3A/B induction was a response to early infection or virion exposure of bystander cells. The observed initial unresponsiveness of urothelium to BKPyV virions may explain why studies of acute infections in mitotically active cell culture systems tend not to observe robust interferon responses (*23*, *51*–*54*). The evidence reported here suggests the innate type I/III interferon response as a critical element of the late (≥21 dpi) viral extrusion microenvironment that induces APOBEC3A, drives the antiviral response, and potentially plays a role in coordinating extrusion [as observed in Paneth cells (*55*)].

The viral apical extrusion process may ultimately clear the BKPyV infection, showing the feasibility of “hit-and-run” carcinogenesis. However, hit and run may not be the best analogy for urothelial carcinogenesis because rather than deriving from the virus itself, the “hit” represents adjacent “friendly fire” derived from paracrine interferons driving collateral APOBEC3A/B damage of the cell’s own chromosomal DNA, leaving them with a form of postinfection stress.

Together, these data suggest episomal BKPyV as a potentially widespread trigger for the initiation of urothelial carcinoma and raise the prospect that the disease may be preventable by antiviral intervention. There are currently no specific small-molecule antivirals or vaccines clinically available for BKPyV. However, as a group both at greater risk of urothelial carcinoma (*56*) and suffering acute BKPyV-associated pathologies (including nephropathy, ureteric strictures, and hemorrhagic cystitis), the renal transplant community eagerly awaits clinical trials for new approaches.

This work calls for a reevaluation of bladder cancer risk factors and consideration of the potential for BKPyV screening approaches as early susceptibility/risk biomarkers in the general population. While APOBEC3A/B mutagenesis drives disease initiation, well-known etiological factors have not been seen to influence urothelial APOBEC3A/B biology (*57*). Here, we report mechanistic evidence supporting episomal BKPyV as a common, persistent renal infection with the means to act as the initiating agent in urothelial carcinoma.

## MATERIALS AND METHODS

### Collection of human urothelial samples

This study was conducted according to the guidelines of the Declaration of Helsinki. The collection and use of ureteric tissue for research were approved by both the Biology Ethics Committee for the University of York (reference JS202208_Study 99-095) and Leeds East National Health Service Research Ethics Committee (reference 99/095). Patient consent was waived, as the ureteric tissue was obtained as anonymous (unlinked), discarded tissue from transplant surgery. Also included in the study was a hemorrhagic cystitis pathology sample (surplus to diagnostic use), which was used, with informed patient consent, under URoBank, a research tissue bank approved by both the Biology Ethics Committee for the University of York (reference JS201611) and Leeds East National Health Service Research Ethics Committee (reference 21/YH/0225).

### Normal human ureteric organ culture

Ureters were dissected to open the vessel exposing the lumen. Two-millimeter sections of full width and thickness ureteric tissue were further dissected and placed into organ culture at an air:liquid interface in Dulbecco’s Modified Eagle’s Medium (DMEM):RPMI 1640 (50:50) supplemented with 5% fetal bovine serum (*58*, *59*). Organ cultures were infected with BKPyV by direct pipetting of 1 × 10^6^ infectious virions onto the luminal surface of the urothelium. After overnight incubation at 37°C, medium volume was increased to submerge the organ cultures and retain stromal viability in long-term culture. This approach maintains a healthy tissue for at least 1 month in culture. At the required point, tissues were fixed in 10% neutral-buffered formalin and processed into paraffin wax using standard histological methods.

### NHU cell culture

Thirty-seven independent NHU cell lines of finite (nonimmortalized) lifespan were used in this study. The NHU cell lines were established as described (*60*) from ureteric tissue. NHU cells were propagated on Cell+ culture plastic (Sarstedt) in keratinocyte serum-free medium (0.09 mM Ca^2+^), supplemented with bovine pituitary extract, recombinant human epidermal growth factor, and cholera toxin. Following expansion through serial subculture, NHU cell cultures were rendered mitotically quiescent and functionally differentiated in medium supplemented with adult bovine serum and [Ca^2+^] elevated to 2 mM, according to well-characterized published methods (*22*, *57*, *61*), and are referred to here as “dNHU cell cultures.” While outside the scope of this report, the organ culture and dNHU cell culture models of normal human urothelium were previously compared in this review (*62*).

Competitive inhibitors of JAKs, which bind to the adenosine 5′-triphosphate–binding domain blocking the kinase’s ability to phosphorylate target proteins, were titrated for equivalent inhibition of IFN-α1–induced STAT2 phosphorylation (100 ng/ml) at 4 hours in dNHU cell cultures and subsequently used at the following concentrations: 2.5 μM peficitinib (Cambridge Bioscience), 2.5 μM pyridone 6 (BioTechne), and 1 μM Ruxolitinib, (Cambridge Bioscience). Where included, JAK inhibitors were added to cultures as a 3-hour pretreatment before infection and were then maintained throughout the time course by medium changes every 48 to 72 hours. Dimethyl sulfoxide was used as a vehicle for the JAK inhibitors and was matched across the three inhibitors and, in the controls, to a final concentration of 0.025% (v/v) in culture medium.

### HBLAK cell culture, CRISPR engineering, subcloning, and validation

The immortalized HBLAK human urothelial cell line (CELLnTEC, RRID:CVCL_JQ59) was cultured according to the published methods (*32*), and CRISPR-Cas9 engineering was performed by Synthego using three guide RNAs (gRNAs) targeting the homologous sequences encoding the catalytic domains of APOBEC3A and APOBEC3B (fig. S7). Synthego confirmed a knockout efficiency of 97% in the delivered pools by polymerase chain reaction (PCR) (fig. S7). For NanoSeq experiments, HBLAK-derived cells were grown and differentiated in CnT-Prime 3D barrier medium (CELLnTEC). HBLAK subcloning was performed by diluting cells to 0.5 cells per 100 μl and then seeding 100 μl of diluted cells into 96-well plates. Plates were checked regularly for cellular growth, and once a well reached 80% confluence, cells were detached using Accutase (MilliporeSigma) and moved to a larger plate or flask (96 → 48 → 12 → 6 → T25 → T75 → T225). During subcloning, HBLAKs were cultured in CnT Prime with 10 μM Rho-associated coiled-coil kinase (ROCK) inhibitor (Y-27632, MilliporeSigma) to promote adhesion and replication until cells reached 80% confluency in a 12-well plate.

Whole-genome sequencing (WGS) was performed on 1 μg of DNA isolated from parental HBLAKs, the non-targeting control (NTC) clone, and the *A3A/A3B*^KO^ clone by the CCR Sequencing Facility on the Illumina NovaSeq 6000. Reads were quality trimmed using fastp, aligned with bowtie2 with the very-sensitive-local option, and deduplicated with Picard. Parental HBLAK variants were called using GATK4 HaplotypeCaller with default parameters. Variants in clones relative to parental HBLAKs were called using GATK4 Mutect2, GATK4 CNV, and Manta (*63*). HBLAKs were validated by comparing single nucleotide variants (SNVs) from WGS data generated for this study against the previous published exome SNVs (*64*). The SNVs (81.8%) originally reported by exome sequencing (*n* = 880) were identical to those detected by WGS, and 16.3% of the SNVs overlapped indels called in WGS. Differences in sequencing platform and analysis method likely explain these differences. The *A3A/A3B*^KO^ clone was further characterized from the WGS data, confirming complete loss of the nucleotide in the first position of the codon for the essential histidine at residues 70 (APOBEC3A) and 273 (APOBEC3B) (fig. S7) (*65*). In addition, on one copy of A3B, there is a 17–base pair (bp) deletion upstream and overlapping the targeted base, as well as a 30-bp deletion at position chr22:38981173, upstream of APOBEC3B, and a 759-bp deletion at position chr22:38957184, upstream of APOBEC3A.

HBLAK protein lysates for Western blot were harvested by directly lysing approximately 1 million pelleted cells into 100 μl of Laemmli reducing sample buffer. Samples were then boiled for 10 min, and 10 μl was run on a NuPAGE 4 to 12% bis-tris gel (Thermo Fisher Scientific) and transferred to a nitrocellulose membrane using standard procedures. Western blot images were captured on a GE Amersham Imager 480. Densitometry quantification of Western blots and deaminase assays was conducted using FIJI (v2.14.0/1.54f) on 16-bit black and white TIFF images with no oversaturated pixels in the quantified bands as confirmed by the Amersham capture software highlighting saturated pixels. APOBEC3 and LT-Ag band density was then normalized to β-tubulin band density.

For HBLAK deaminase assays, a 6-hour treatment with PMA (25 ng/μl; Invivogen) was used as a positive control to induce APOBEC3B. One million cells were pelleted and then resuspended in 100 μl of HED buffer (25 mM Hepes, 5 mM EDTA, 10% glycerol, 1 mM dithiothreitol, and 1 EDTA-free proteasome inhibitor tablet). Cells were sonicated with a PIXUL sonicator with default settings for 20 min, centrifuged at 16,000 Relative Centrifugal Force (RCF) for 20 min at 4°C, and then transferred to fresh tubes. Protein content was quantified by 280-nm spectrophotometry, and lysates were normalized to 2 μg/μl. Cleared lysate (16.5 μl) was used for deaminase reactions in 25 μl of ribonuclease A (200 ng/ml; Thermo Fisher Scientific), 2 μl of 10× Uracil-DNA glycosylase (UDG) buffer [New England Biolabs (NEB)], 0.25 μl of UDG enzyme (NEB), and 1 μl 4 μM fluorescein-labeled TTCA-containing oligo. Deaminase reactions were run for 18 hours at 37°C. Two microliters of 1 M NaOH was added to each sample and boiled for 10 min, and then 22 μl of formamide buffer was added and boiled for an additional 5 min. Reactions were resolved using a 15% Tris/Borate/EDTA (TBE)-urea gel (Thermo Fisher Scientific) and imaged on a GE Amersham Imager 480.

### BKPyV infection

BKPyV was first isolated in 1970, and that research strain was named after the lead author, “Gardner” (*33*); subsequently, a derived strain, “Dunlop,” became widely established (*66*). BKPyV Dunlop strain was expanded for use in renal proximal tubule epithelial cell cultures, which were scrape harvested at 14 dpi, sonicated, and frozen as aliquots at −80°C. MOI was calculated by fluorescent focus unit assay using IncuCyte ZOOM analysis (Essen BioScience, Ann Arbor, MI, USA), as previously reported (*67*). All dNHU cell cultures in this study were infected with 0.45-μm filtered BKPyV-containing medium at an MOI equivalent to 0.01 in RPTE cells, but which varies between donors. Cells were exposed to virus for 3 to 4 hours at 37°C before virus-containing medium was removed and cultures continued.

BKPyV Gardner strain was produced by infection of 293TT cells as described (*68*). HBLAK-derivative cells were grown to 100% confluency in 12-well plates and differentiated in CnT-Prime 3D barrier medium (CELLnTEC) for 7 days. Gardner at 0.5 MOI and mock infections were performed in triplicates as previously described (*16*). Genomic DNA and protein were isolated from biological replicates on days 1, 7, 15, and 25 postinfection or until confluency reached 50%. Genome copies of BKPyV were determined using quantitative PCR (qPCR) as previously described (*69*).

### mRNA analysis

Total RNA was collected in TRIzol reagent (Invitrogen). Samples from mock- and BKPyV-infected dNHU cell cultures at 21 dpi were selected for mRNA-seq using the Illumina NovaSeq 6000 generating 150-bp paired-end reads (Novogene UK, Cambridge, UK). All mRNA-seq data have been deposited at SRA PRJNA1199925. Following standard quality control, gene-level expression values in TPM were derived against the Gencode v43 human transcriptome using kallisto v0.46.1 (*70*). Differentially expressed genes were identified using the Sleuth v0.30.0 (*71*) implementation of the likelihood ratio test (LRT), accounting for matched genetic backgrounds and generating Benjamini-Hochberg–corrected *q* values. APOBEC3A editing of mRNA was assessed using ApoTrack (*25*).

Gene set enrichment analysis (GSEA) (*72*) was performed using π values calculated as described elsewhere (*73*)

$$\pi ={\text{log}}_{2}\text{fold change}\;\left(\text{TPM}+1\right)\times -{\text{log}}_{10}\text{LRT}q$$

(derived from the Sleuth LRT *q* values) and the preranked list feature implemented in the Python package GSEApy (0.10.2, available at https://github.com/zqfang/GSEApy). All genes with a π value of 0 were removed from the preranked list, as they were effectively unranked. The ranked list of genes was run against the Molecular Signatures Database collection (msigdb.v2023.1.Hs.symbols.gmt).

For analysis of the BKPyV Dunlop transcriptome, sequences (derived from BKPyV reference; GenBank NC_001538.1) were appended to the human transcriptome to generate “relative TPMs” as a measure of viral transcript abundance. Reads were also aligned to the human (GRCh38) and BKPyV reference genome assemblies with HISAT2 v2.2.0 (*74*).

### Protein lysate collection and Western blotting

NHU cell cultures were scrape harvested in a lysis buffer containing 0.2% (v/v) protease inhibitors (Protease Inhibitor Cocktail set III, Calbiochem). Lysis buffer comprised 25 mM Hepes-KOH (pH 7.5), 10% glycerol, 150 mM NaCl, 0.5% Triton X-100, and 1 mM ethylenediaminetetraacetic acid. Lysates were sonicated and centrifuged at 13,000*g*. A Bradford protein assay was used to normalize loading into Western blots, and mitogen-activated protein kinase 7/β-actin densitometry was used as a housekeeping protein to check normalization by protein concentration had been successful. Fifty micrograms of protein lysate per lane was resolved on NuPAGE gels using the Novex electrophoresis system (Invitrogen) at 200 V. Electrotransfer to low fluorescence polyvinylidene difluoride membranes (Millipore) was completed in a tris-glycine buffer at 20 V for 2 hours at 4°C before appropriate blocking. Primary antibodies (detail provided in table S1) were applied at 4°C overnight. Membranes were labeled with the appropriate IRDye-conjugated secondary antibody (LI-COR) and visualized by epifluorescent infrared illumination at 700 and/or 800 nm using the Odyssey Sa scanner and software (LI-COR). Densitometry was performed using Image Studio Lite Ver 5.0 software (LI-COR).

### Nanorate sequencing

Genomic DNA was extracted from cultures of NHU cells using NucleoSpin Tissue spin columns (Macherey-Nagel). For the bulk NanoSeq, three conditions were analyzed: mock- and BKPyV-infected cultures, time matched at 21 dpi, and BKPyV-infected cultures at the visually assessed lethal time LT_50_ (i.e., the time where 50% of the culture had been lost to infection and was observed as bare plastic). For the bulk NanoSeq, the mean LT_50_ was 44.3 days (range = 30 to 53).

For the FACS-NanoSeq, mock- and BKPyV-infected cultures from three donors were harvested at time-matched points based on the visually assessed lethal time “LT_10_” (i.e., the time where 10% of the culture had been lost to infection) in the BKPyV-infected cultures. For the FACS NanoSeq, the mean LT_10_ was 72.6 days (range = 36 to 116).

The NanoSeq method was used as described in the original manuscript (*26*) and further detailed in an online protocol (*75*). Briefly, DNA was digested using HpyCH4V (NEB) before A-tailing using Klenow Fragment (3′ → 5′ exo-; NEB) in the presence of dideoxy bases (“ddBTPs”; preventing any fragment subject to potentially error-prone nick repair from subsequent amplification and minimizing error introduction into final libraries). IDT xGen CS adapters, which contain unique molecular identifiers, were then ligated to fragment ends, and resulting fragments were quantified. A total of 0.08 fmol was input into libraries generated with 13 cycles of PCR. The undiluted (3 fmol of input) libraries used for subtraction of germline mutations were generated from the control sample for each individual donor–derived culture.

The NanoSeq method uses an archaeal proofreading DNA polymerase (NEB Q5 DNA polymerase) in the library preparations. Archaeal DNA polymerases contain a uracil-binding pocket that causes the enzyme to stall when confronted with a uracil (*76*). This is particularly relevant to the study of APOBEC mutational signatures, since active APOBEC deamination of cytosine forms uracil lesions in the genome before the formation of a true mutation. Using an archaeal DNA polymerase in NanoSeq library preparation prevents uracil lesions from contributing to the signature. Thus the mutational signatures reported here represent true mutations and not simply deamination lesion sites.

Pooled libraries were sequenced on an Illumina NovaSeq 6000 or NovaSeq X machine at Genewiz (from Azenta Life Sciences). For the bulk NanoSeq test samples, a mean of 321 million reads per sample was analyzed with a mean duplicate rate of 0.79 (the theoretical optimal duplicate rate is 0.81). For the FACS-NanoSeq test samples, a mean of 224 million reads per sample was analyzed with a mean duplicate rate of 0.81. All NanoSeq data have been deposited at SRA PRJNA1199925.

### NanoSeq bioinformatics

A custom pipeline was created using Snakemake v7.22.0 to execute the NanoSeq v3.2.1 bioinformatics workflow using the default parameters in a Slurm environment. The Snakemake pipeline includes sequencing quality control, full processing, and analysis of sequenced NanoSeq samples (FASTQ to VCF), contamination checks, and efficiency estimations.

Variants in VCF files were annotated using VEP v107 (*77*). The plugin split-vep from BCFtools v1.19 (*78*) was used to tabulate the VEP-annotated VCFs, returning the annotation with the most severe consequence (option: -s worst) in cases where more than one gene was associated with a variant. A custom Python script was used to identify the quintuplet base context in the GRCh38 reference genome sequence surrounding each single-nucleotide variant.

The read bundle conformation section of the Perl script efficiency_nanoseq.pl from the NanoSeq bioinformatics workflow was modified to return read bundle information for the whole genome, as opposed to the default of chromosome 1 only. A custom R script was used to count the number of OK read bundles per sample (OK = at least two reads from two original strands). The number of mutations was divided by the number of OK read bundles per sample and multiplied by 300 (estimation for base pairs sequenced per read bundle) to estimate the number of mutations per gigabase of DNA sequenced.

Mutations of the BKPyV genome during infection were detected using the NanoSeq sequencing data but via a custom scripting pipeline. Reads were aligned to the BKPyV genome (Dunlop strain, GenBank: NC_001538.1) using minimap2 v2.26 (*79*) with the short read preset, retaining only mapped reads. Alignment maps were processed using Picard v2.20.2 (“Picard Toolkit,” 2019, Broad Institute, GitHub repository, https://broadinstitute.github.io/picard/; Broad Institute) and SAMtools v1.20 (*78*), including markdup to annotate read bundles. Viral mutations were called using SAMtools mpileup, removing indels, requiring mapping and base quality scores of ≥30, and removing variants with a frequency of ≥0.05. The SBS/DBS signatures were analyzed using the “Catalog” input for the “signal” workflow (*80*) for mutational signature analysis (https://signal.mutationalsignatures.com/).

### Immunoperoxidase labeling

Paraffin wax–embedded normal human ureteric organ cultures and excess diagnostic tissue from a patient with BKPyV-associated hemorrhagic cystitis were sectioned at 5 μm, and once dewaxed, sections were incubated in hydrogen peroxide to neutralize endogenous peroxidase activity. Heat-mediated antigen retrieval was performed by boiling for 10 min in 10 mM citric acid buffer (pH 6) or 1 mM EDTA buffer (pH 8) to enhance detection by primary antibodies including positive and negative specificity controls (detail provided in table S1), which were applied overnight at 4°C. Signal amplification was achieved with anti-mouse and anti-rabbit ImmPRESS Excel staining kits (Vector Laboratories), used according to the manufacturer’s instructions. Labeled sections were counterstained with Mayer’s hematoxylin, dehydrated, and mounted in DPX Mountant (Thermo Fisher Scientific).

### Indirect immunofluorescence

NHU cell cultures on glass 12-well slides were fixed in methanol:acetone (1:1) 30 s, air dried, and stored frozen. Primary antibodies (detail provided in table S1) were applied overnight at 4°C. Unbound primary antibodies were removed by washing in phosphate-buffered saline (PBS) and secondary antibodies [goat anti-mouse immunoglobulin (Ig) Alexa Fluor 488 (AF488) and goat anti-rabbit Ig AF594, Molecular Probes] were applied for 1 hour at ambient temperature. Slides were washed in PBS, with Hoechst 33258 (0.1 μg/ml) added to the penultimate wash, before mounting in ProLong Gold Antifade Mountant (Thermo Fisher Scientific) and visualization by epifluorescence on a BX60 microscope (Olympus).

### Fluorescence-activated cell sorting

Differentiated NHU cell sheets were lifted from culture plastic by incubation in Dispase II [0.5% (w/v) in Dulbecco’s PBS]. Harvested sheets were disaggregated into single-cell suspensions by sequential 5-min incubations in 0.1% EDTA and Trypsin Versene. Single cells were fixed in methanol:acetone (50:50) with constant agitation for 30 s before diluting out the solvents and pelleting of cells by centrifugation (600*g*). Fixed cells were resuspended in primary antibodies (detail provided in table S1) for incubation overnight in microcentrifuge tubes at 4°C on a blood tube rotator. After a single wash, cells were incubated in goat anti-rabbit AF555 and goat anti-mouse AF488 (Thermo Fisher Scientific) for 1 hour at ambient temperature. After one final wash, cells were analyzed and sorted into four subpopulations with half log separation between sort gates using a Beckman Coulter MoFlo Astrios Eq [sort mode = purify 1 cell per drop; AF555 excited with 561-nm laser 579/16 detection filter; AF488 excited with 488-nm laser 526/52 detection filter] hosted in the Bioscience Technology Facility at York. Acquisition software was Beckman Coulter Summit v6. Sort purity and yield were verified with beads before the sort and the sort drop monitored throughout the sort duration. DNA was extracted from the subpopulations using a NucleoSpin Tissue XS kit (Macherey-Nagel). A mean of 307,281 cells were sorted per donor for each condition (mock- and BKPyV-infected). Analysis was performed using Dotmatics FCS Express v7.

### Cancer cohort DNA sequencing data

The Cancer Genome Atlas (TCGA) project recently released WGS data for a cohort of MIBC samples with matched mRNA-seq data (*n* = 402) (*21*), which has previously been used to derive consensus molecular subtypes (*81*). BAM files for MIBC and matched normal samples (preferentially and predominantly blood-derived but adjacent normal where blood was not sequenced) were downloaded from the database of Genotypes and Phenotypes (dbGaP) reference phs000178 under project code 25297. For consistency with previously reported processing of whole-exome data (*21*), single-nucleotide variants were called with MuTect2 (GATK v4.3.0.0) using TCGA reference files for the GRCh38 genome (GRCh38.d1.vd1), germline variants (af-only-gnomad.hg38), and panel of normals (MuTect2.PON.5210). GATK v4.1.0.0 FilterMutectCalls was used to annotate variant quality, with variants passing filtering criteria kept. Variants were further filtered on the basis of depth using BCFtools v1.19, retaining variants with sequencing depth of ≥10 and ≤the median depth plus 1.5 × interquartile range. In catalog format, the SBS (in triplet and pentanucleotide contexts) and DBS signatures were analyzed using the signal workflow (*80*) for mutational signature analysis (https://signal.mutationalsignatures.com/). Since TCW trinucleotides make up 8.4% of the human genome (GRCh38), for analysis of the APOBEC3A versus APOBEC3B characteristics of the signatures, samples with <16.8% of mutations derived from TCW were removed. Sample “TCGA-DK-A6AW” was also removed because of an atypical number of T[C>A]T mutations, and this filtering retained 348 samples (87%) with >2-fold enrichment of APOBEC-characteristic TCW mutations than expected by chance. One luminal nonspecified MIBC sample “TCGA-DK-A3IT” has previously been reported to have the BKPyV genome integrated into that of the host (*20*).

### Statistical analysis

Data were assessed for statistical significance using Prism v10 software (GraphPad). On all graphs, statistical *P* or *q* value significance is represented as follows: *<0.05, **<0.01, and ***<0.001. The abbreviation “ns” denotes that an effect was not statistically significant.
