# Supplementary material for: Virus-induced APOBEC3 transmutagenesis in bladder cancer initiation
Source: Sci Adv. 2025 Dec 3;11(49):eaea6124. doi: 10.1126/sciadv.aea6124 (PMC12674112; doi:10.1126/sciadv.aea6124)
Supplement: Supplementary file 1 — Figs. S1 to S13 Table S1 [file sciadv.aea6124_sm.pdf]

Science Advances
AAAS

Supplementary Materials for

Virus-induced APOBEC3 transmutagenesis in bladder cancer initiation

George H. Hatton *et al.*

Corresponding author: Simon C. Baker, simon.baker@york.ac.uk

Sci. Adv. **11**, eaea6124 (2025)
DOI: 10.1126/sciadv.aea6124

This PDF file includes:

Figs. S1 to S13
Table S1

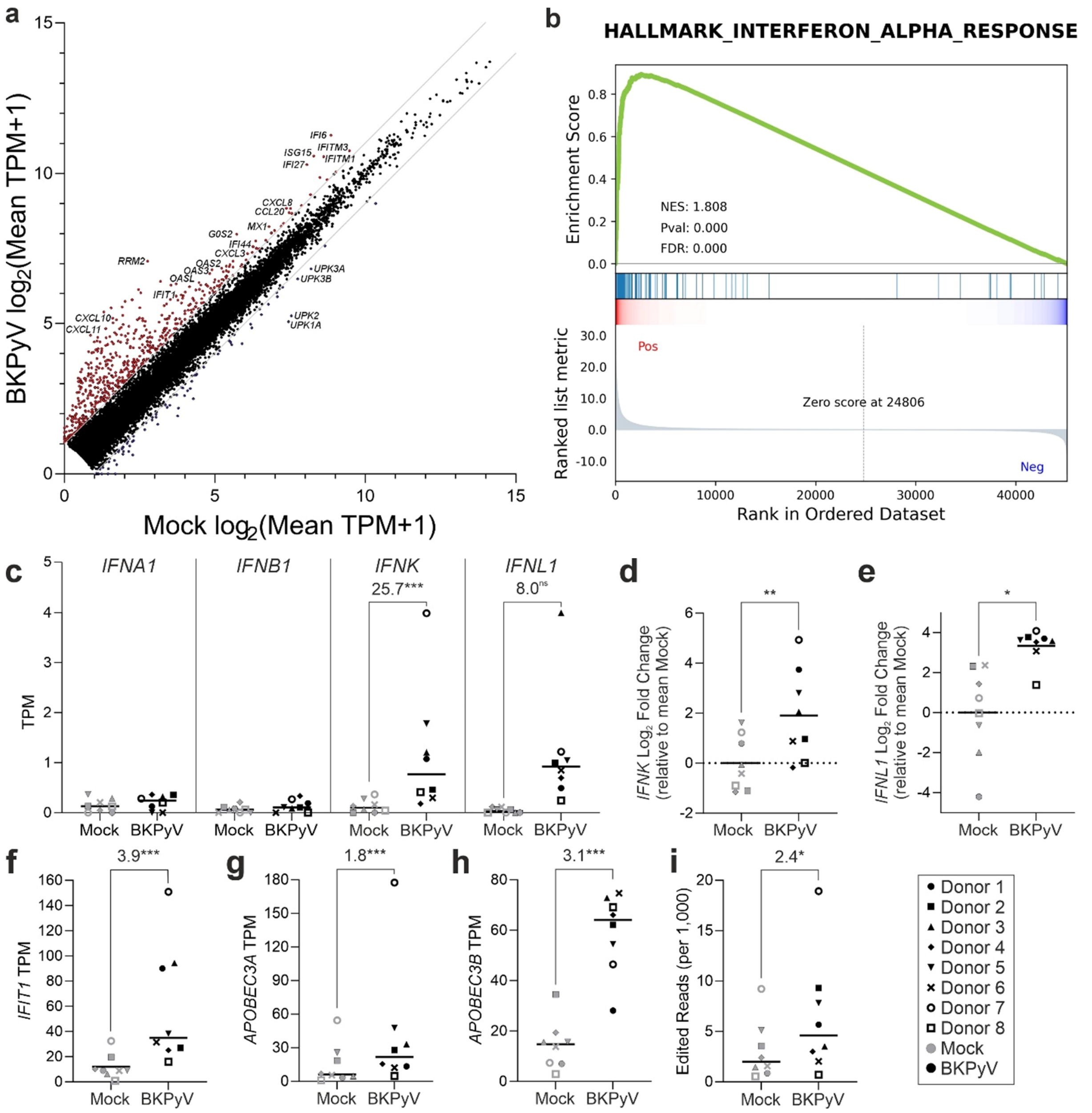

Fig. S1 – **a** Scatter plot of $\log_2$ transformed mean transcripts per million (TPM) values for Mock and BKPyV infected differentiated normal human urothelial cell (dNHU) cultures at 21 days post infection (dpi). Amongst the highly expressed and further induced genes many were part of the interferon response (including *IFIT1*, *IFITM1*, *IFITM3*, *IFI6*, *IFI27*, *ISG15*, *OAS2*, *OAS3* and *OASL*). By contrast in the high expression but down regulated genes were superficial urothelial differentiation markers known as the uroplakins (*UPK* genes) which decorate the luminal surface of the barrier-forming tissue. Light grey lines show the 2-fold change cutoffs with upregulated genes given red datapoints and downregulated genes denoted in blue. A specifically selected subset of the statistically significantly changing genes are labelled. **b** Gene set enrichment analysis (72) of mRNA-sequencing data from BKPyV infection of dNHU cultures at 21 dpi revealed significant

induction of the “Hallmark Interferon Alpha Response” gene set. **c** mRNA-sequencing of dNHU cultures at 21 dpi with BKPyV Dunlop showed no change in *IFNA1* or *IFNB1*; however, increases in *IFNK* (type I interferon) and *IFNL1* (type III interferon) transcripts were observed (fold changes and statistical significances are shown on the graphs). **d-e** Due to the low TPM values observed for interferon transcripts, the induction of *IFNK* and *IFNL1* transcripts were confirmed by reverse transcription quantitative polymerase chain reaction. **f-h** Transcripts for the canonical interferon stimulated gene or “ISG” *IFIT1* and *APOBEC3A* and *APOBEC3B* all increased significantly during BKPyV infections compared to mock cultures at 21 dpi. **i** ApoTrack analysis (25) of mRNA hairpin editing by APOBEC3A revealed a significant mean 2.4-fold increase at 21 dpi with BKPyV Dunlop in dNHU cultures (ratio paired t test).
For all panels in this figure, n=8 independent cell lines. The legend in the bottom right of the figure applies to panels **c**-**i**.

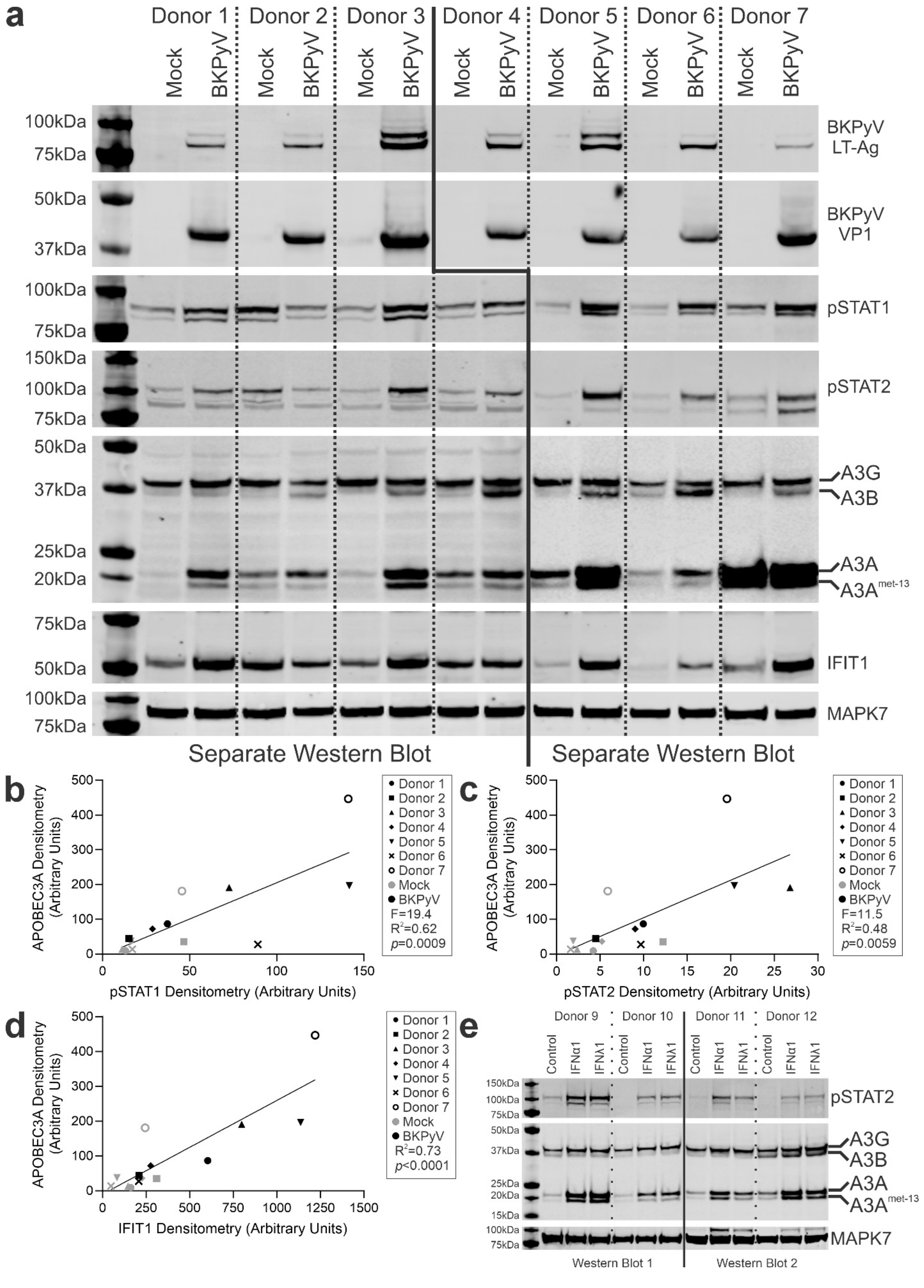
a
Donor 1
Donor 2
Donor 3
Donor 4
Donor 5
Donor 6
Donor 7
Mock
BKPyV
100kDa
75kDa
50kDa
37kDa
150kDa
25kDa
20kDa
BKPyV LT-Ag
BKPyV VP1
pSTAT1
pSTAT2
A3G
A3B
A3A
A3A met-13
IFIT1
MAPK7
Separate Western Blot
Separate Western Blot
b
APOBEC3A Densitometry (Arbitrary Units)
pSTAT1 Densitometry (Arbitrary Units)
Donor 1
Donor 2
Donor 3
Donor 4
Donor 5
Donor 6
Donor 7
Mock
BKPyV
F=19.4
R²=0.62
p=0.0009
c
APOBEC3A Densitometry (Arbitrary Units)
pSTAT2 Densitometry (Arbitrary Units)
F=11.5
R²=0.48
p=0.0059
d
APOBEC3A Densitometry (Arbitrary Units)
IFIT1 Densitometry (Arbitrary Units)
R²=0.73
p<0.0001
e
Donor 9
Donor 10
Donor 11
Donor 12
Control
IFNα1
IFNλ1
15kDa
Western Blot 1
Western Blot 2

Fig. S2 – Western blotting of differentiated normal human urothelial cell (dNHU) cultures at 21 days post infection (dpi) with BKPyV (Dunlop strain; Multiplicity of Infection = 0.1). **a** Images relating to the densitometry analysis shown in **Fig. 1C** from 7 independent samples matched to those collected for the mRNAseq data and the NanoSeq data in **Fig. 2**. Targets include Large T antigen (LT-Ag), Viral capsid major Protein 1 (VP1), phosphorylated forms of Signal Transducer and Activator of Transcription 1 and 2 (pSTAT1 and pSTAT2), APOBEC3 enzymes, interferon-induced protein with tetratricopeptide repeats 1 (IFIT1) and the house-keeping protein mitogen-activated protein kinase 7 (MAPK7). **b** Linear regression analysis suggested the frequency of STAT1 phosphorylation significantly predicted APOBEC3A protein expression (n=7 independent cell lines). **c** Linear regression analysis suggested the frequency of STAT2 phosphorylation significantly predicted APOBEC3A protein expression (n=7 independent donor NHU cell lines). **d** Correlation analysis suggested protein expression of (the canonically interferon stimulated) IFIT1 was significantly associated with expression of APOBEC3A (n=7 independent cell lines). **e** Western blotting suggested exogenous addition of both type I (IFNα1; 100ng/mL) and type III (IFNλ1; 100ng/mL) interferons to dNHU cultures for 72 hours were capable of inducing APOBEC3A protein expression but highlights inter-donor variance in response. n=4 independent NHU cell lines.

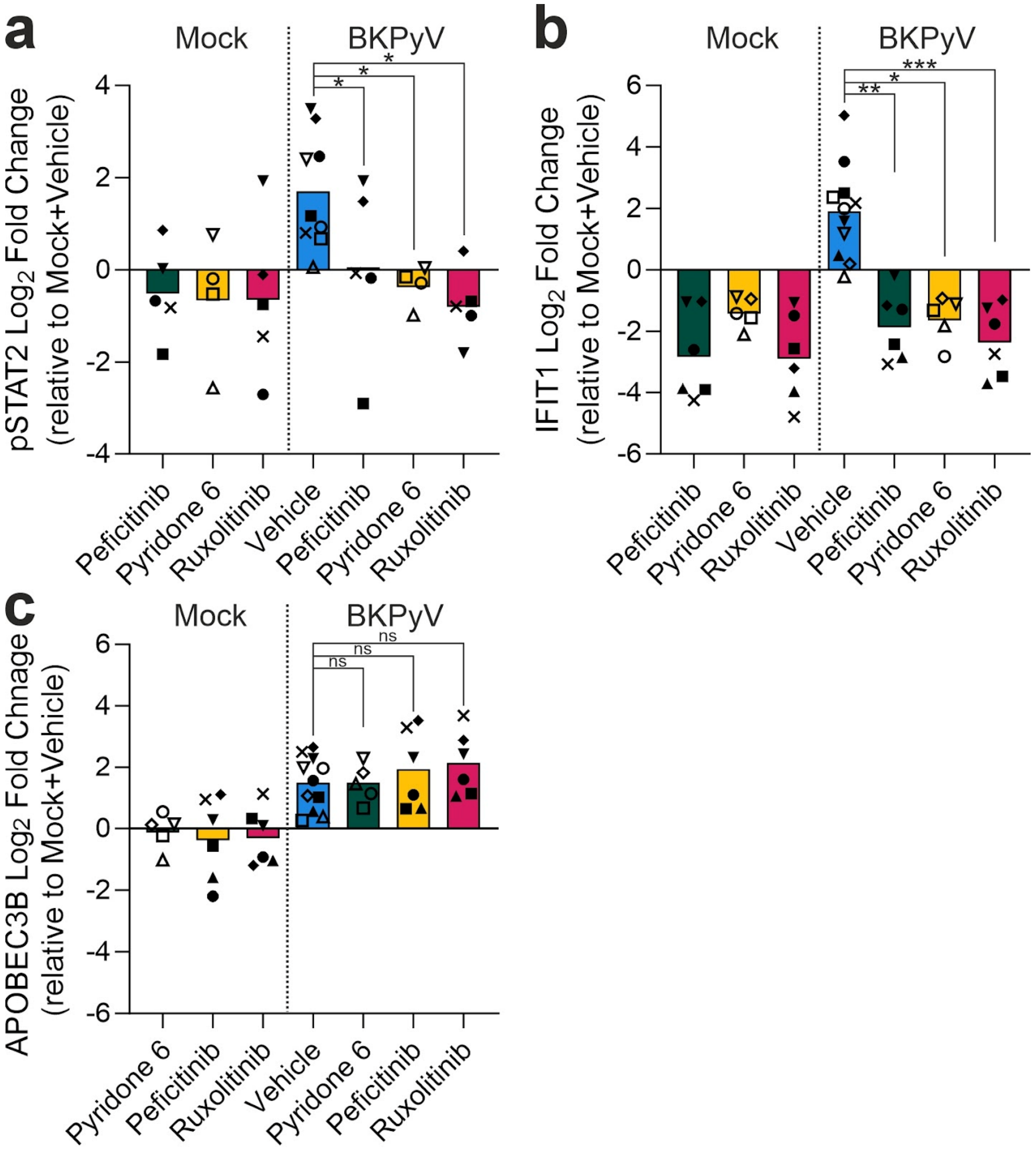

Fig. S3 – Inhibition of innate type I/III interferon signalling by competitive JAK inhibitors; 2.5µM Peficitinib, 2.5µM Pyridone 6 or 1µM Ruxolitinib (n≥5 independent NHU cell lines). JAK inhibition led to a statistically significant decrease in phosphorylation of Signal Transducer and Activator of Transcription 2 (pSTAT2), and significantly reduced expression of interferon-induced protein with tetratricopeptide repeats 1 (IFIT1). By contrast with APOBEC3A (**Fig. 1E**), no change was observed in the expression of APOBEC3B.

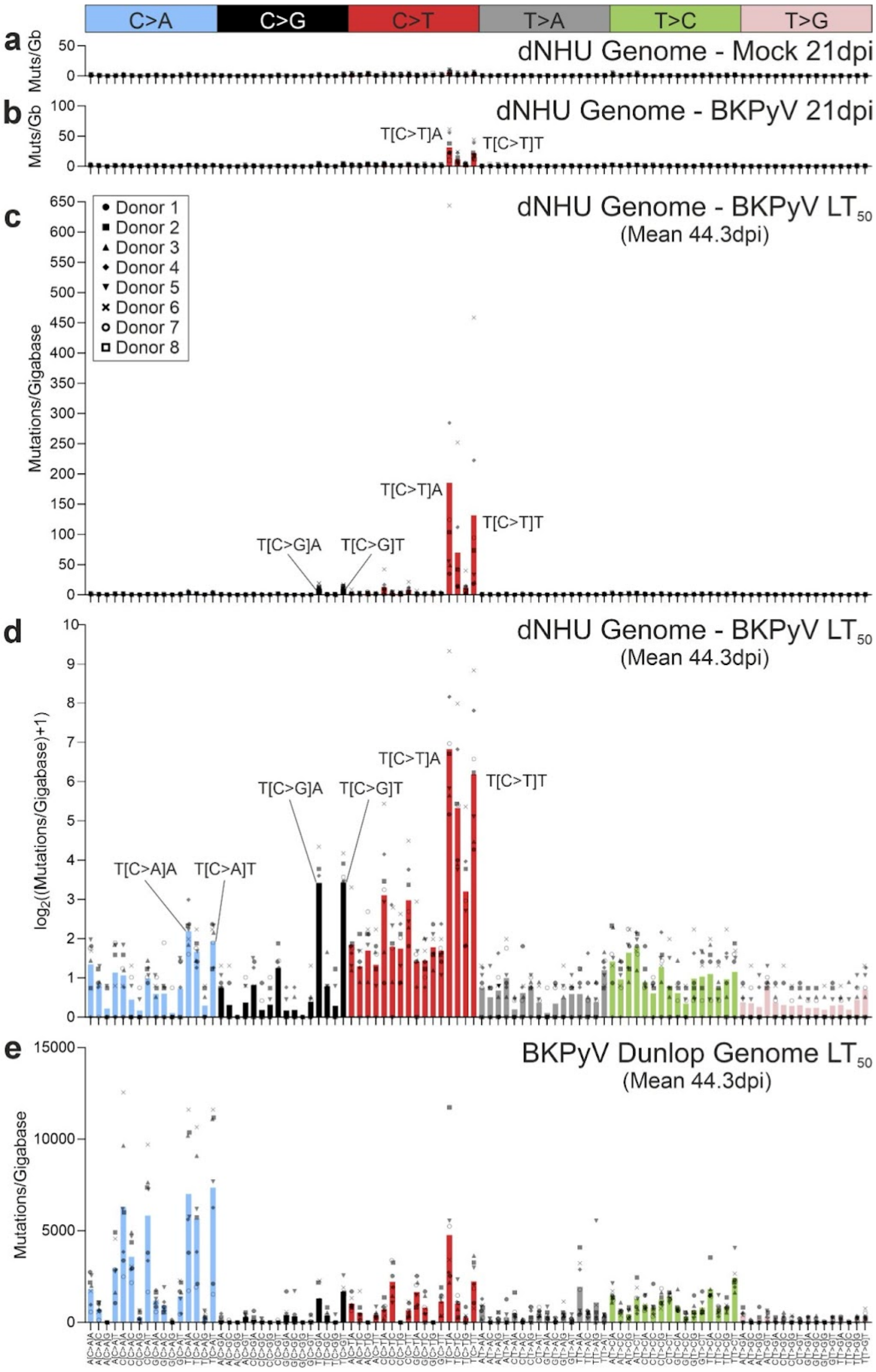

Fig. S4 – Nanorate sequencing (NanoSeq) of mock and BKPyV (Dunlop; MOI=0.01) infected differentiated normal human urothelial cell (dNHU) cultures was used to derive mutational signatures at 21 days post infection (dpi; panels **a** and **b**) and **c** a timepoint selected where cell culture density reached 50% due to lytic infection (“LT_{50}”). Panel **d** shows the data from panel **c** log_2 transformed to highlight the SBS13 mutations (T[C>A]W and T[C>G]W, where W=A/T). The plot labels highlight the APOBEC3A/B T<u>**C**</u>W mutations used in other analyses. **e** Mutations observed in the BKPyV Dunlop genome during infections of dNHU cultures. Plots show the individual donors with the unique point identifiers shown in the legend of panel **c**.

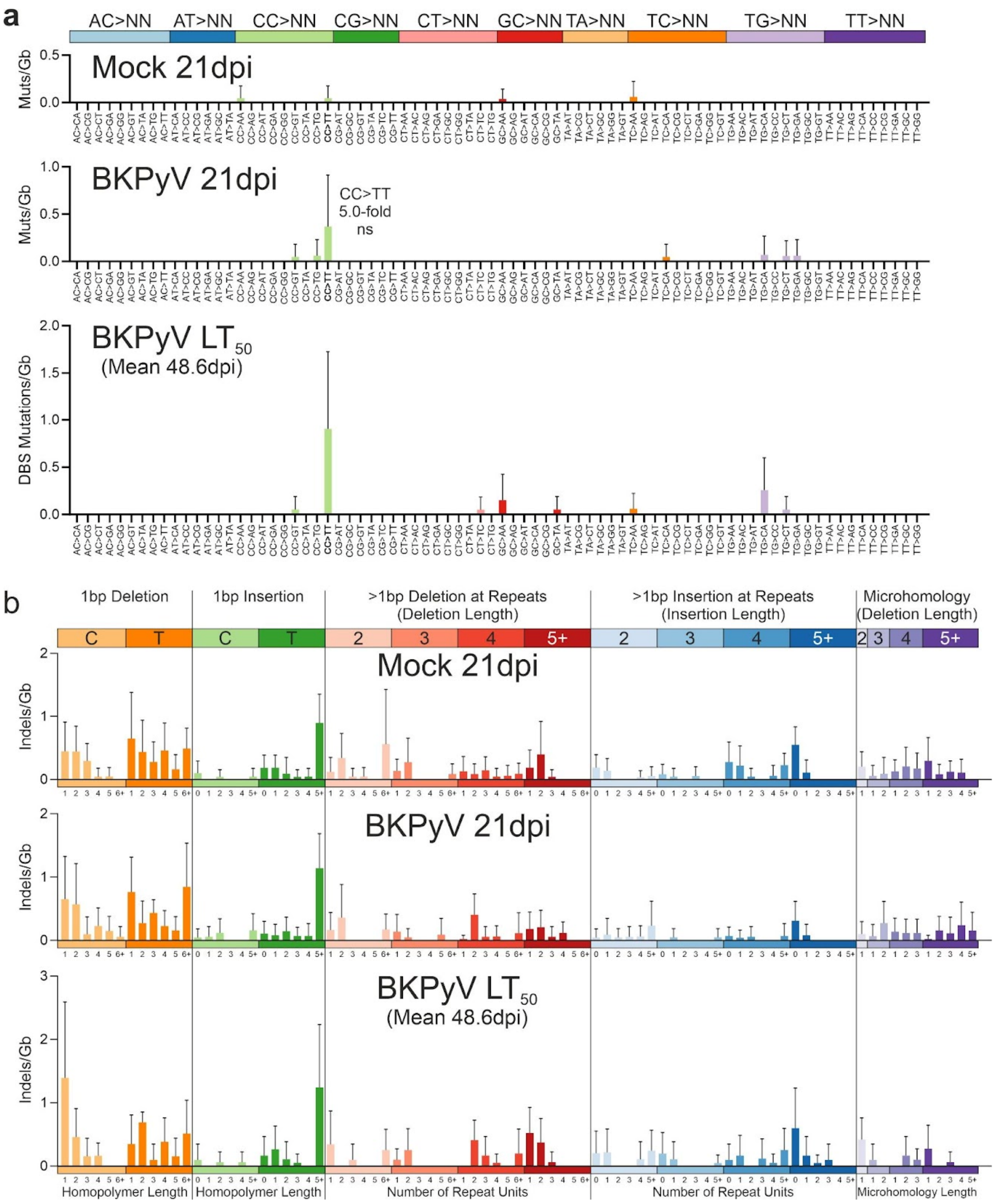

Fig. S5 – Extended Mutational Signatures. **a** Double base substitution (DBS) mutational signatures from BKPyV infection expressed as the count for each mutation type divided by the number of gigabases from the NanoSeq library that was sequenced. The graphs highlight an increase in the frequency of CC to TT mutations that was not significant due to the low detection rate and donor variance. The pattern does show similarity to the Catalogue of Somatic Mutations in Cancer (COSMIC) DBS11 signature which has been associated with APOBEC SBS2 and SBS13 in cancer genomes (19). **b** Insertion/Deletion (ID) mutational signatures from BKPyV infection expressed as the count for each mutation type ("Indels") divided by the number of gigabases (Gb) from the NanoSeq library that was sequenced. No significant changes were observed; however, at the timepoint where cell culture density reached 50% due to lytic infection ("LT_{50}") an ID signature of single bp C deletions in isolated bases and potentially T insertions in long homopolymers runs similar to that described prospectively for APOBEC3A overexpression may have been developing (29).

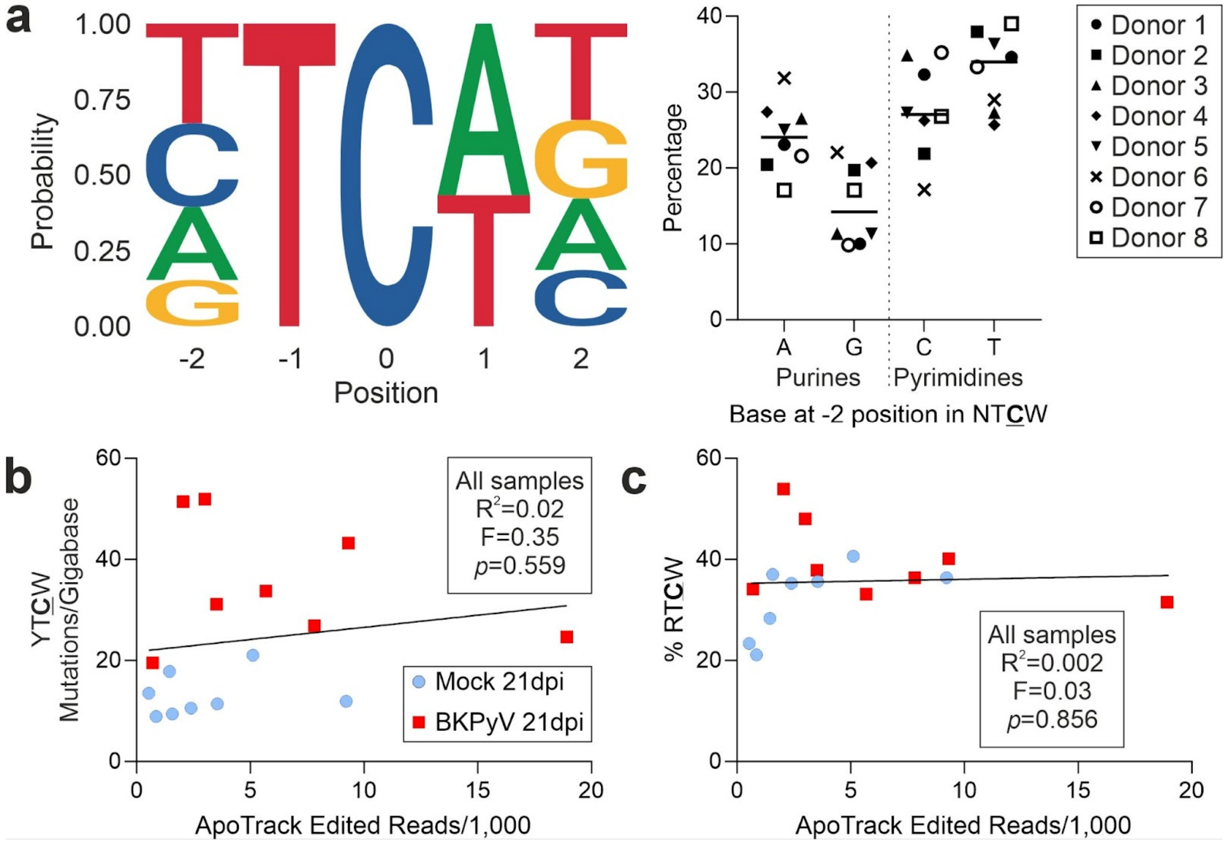

Fig. S6 – Bulk differentiated normal human urothelial cell (dNHU) culture NanoSeq and mRNA analysis at 21 days post infection (dpi) with BKPyV Dunlop. **a** The 5bp footprint of APOBEC signature T<u>C</u>W (where W=A/T) cytosine mutations showed the mean purine percentage at the -2 position "RT<u>C</u>W" (where R=A/G), for all 8 donors at 21 dpi was 39.4%. However, donors 4 and 6 which had the highest numbers of T<u>C</u>W mutations (**Fig. 2D**) and highest APOBEC3B/APOBEC3A protein ratio (**Fig. 2E**) had a mean RT<u>C</u>W of 51.0%, suggestive of APOBEC3B dominance. The rest of the donors had a mean RT<u>C</u>W of 35.5%, suggesting mixed APOBEC3A > APOBEC3B activity.

Simple linear regression was used to test if APOBEC3A mRNA editing, assessed as ApoTrack Edited Reads, significantly predicted either **b** the APOBEC3A-character YT<u>C</u>W DNA mutations formed per donor or **c** a decreasing % of RT<u>C</u>W mutations. In neither case did mRNA editing characteristic of APOBEC3A significantly predict DNA editing of the type associated with APOBEC3A. Legend in panel **b** applies to panel **c** as well.

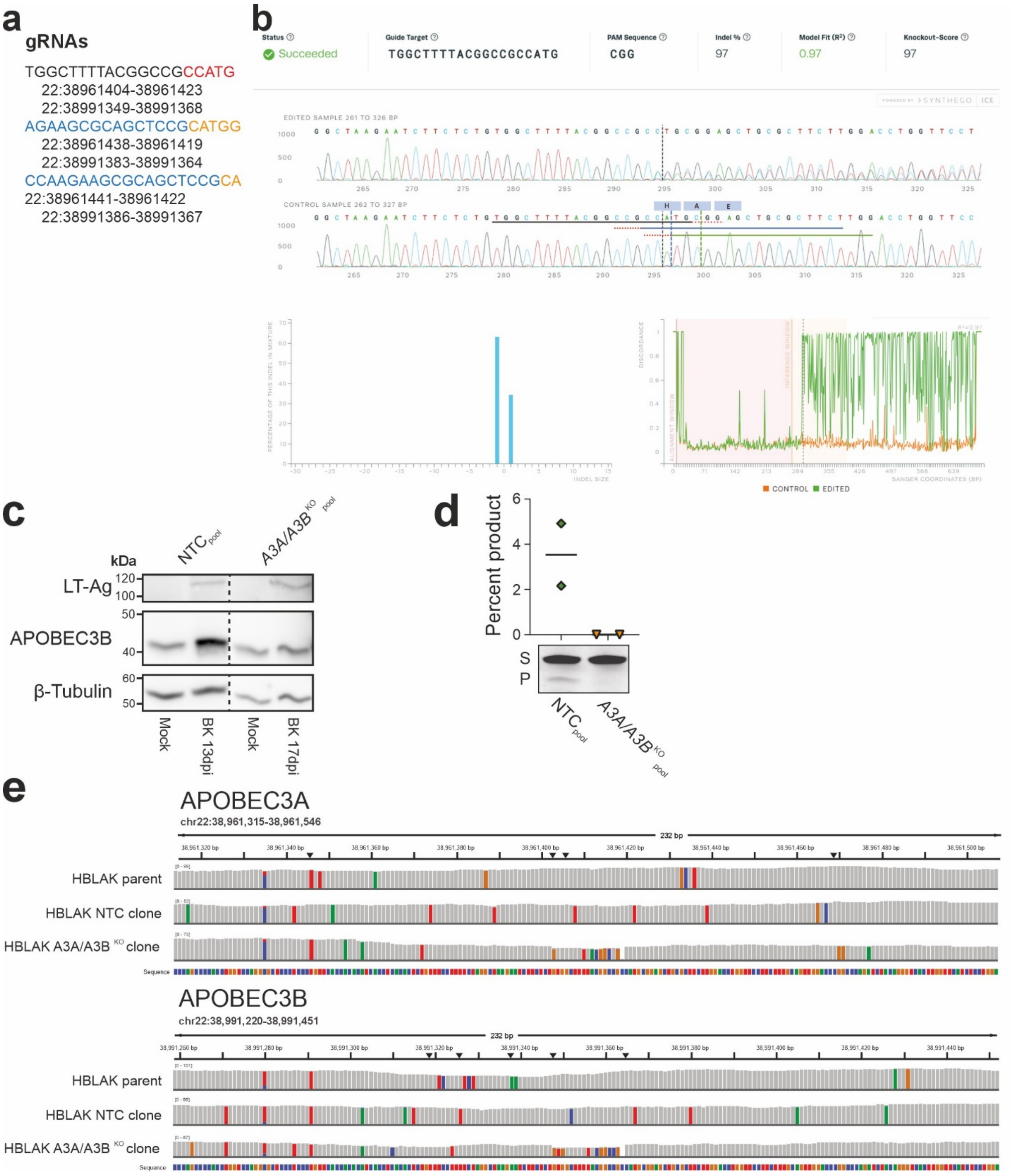

Fig. S7 – Engineering of APOBEC3A and APOBEC3B functional deletion in the HBLAK urothelial cell line. **a** gRNAs used for CRISPR-Cas9 engineering of HBLAK cells. Overlapping sequences between the gRNAs are highlighted in blue, and orange/red with orange being the reverse compliment sequence of red sequences. **b** Engineering report from Synthego including Sanger sequencing of homologous region between *APOBEC3A* and *APOBEC3B* showing the frameshifted mutation in the cultures compared to the control cells prior to cloning and calculated

editing efficiency of 97% on both loci. gRNA targets are highlighted with solid blue, black, and green lines with PAM sequences highlighted by red dotted lines. Cut sites are shown with vertical dashed lines for each gRNA. **c** Western blotting of the HBLAK functional knockout culture pools prior to cloning confirmed viral infection by detection of Large T antigen (LT-Ag) protein. APOBEC3B was induced by non-targeted control cells in response to BKPyV infection but not in the *A3A/A3B*^{KO}. APOBEC3A was not detectable in HBLAK cell lysates (n=2; exemplar western blot shown). **d** Deaminase activity assays confirmed the knockout of APOBEC3-function in PMA-treated *A3A/A3B*^{KO} HBLAK cell pools prior to cloning, with the exemplar gel showing bands for substrate "S" in both sample-types and deamination-dependent product "P" only in NTC lysates (n=2). **e** Illumina whole genome sequencing of parental HBLAKs, non-targeting control (NTC) clone, and *A3A/A3B*^{KO} edited clone showing the absence of the targeted first position of the codon for the essential histidine (65) at residues 70 in A3A and 273 in A3B.

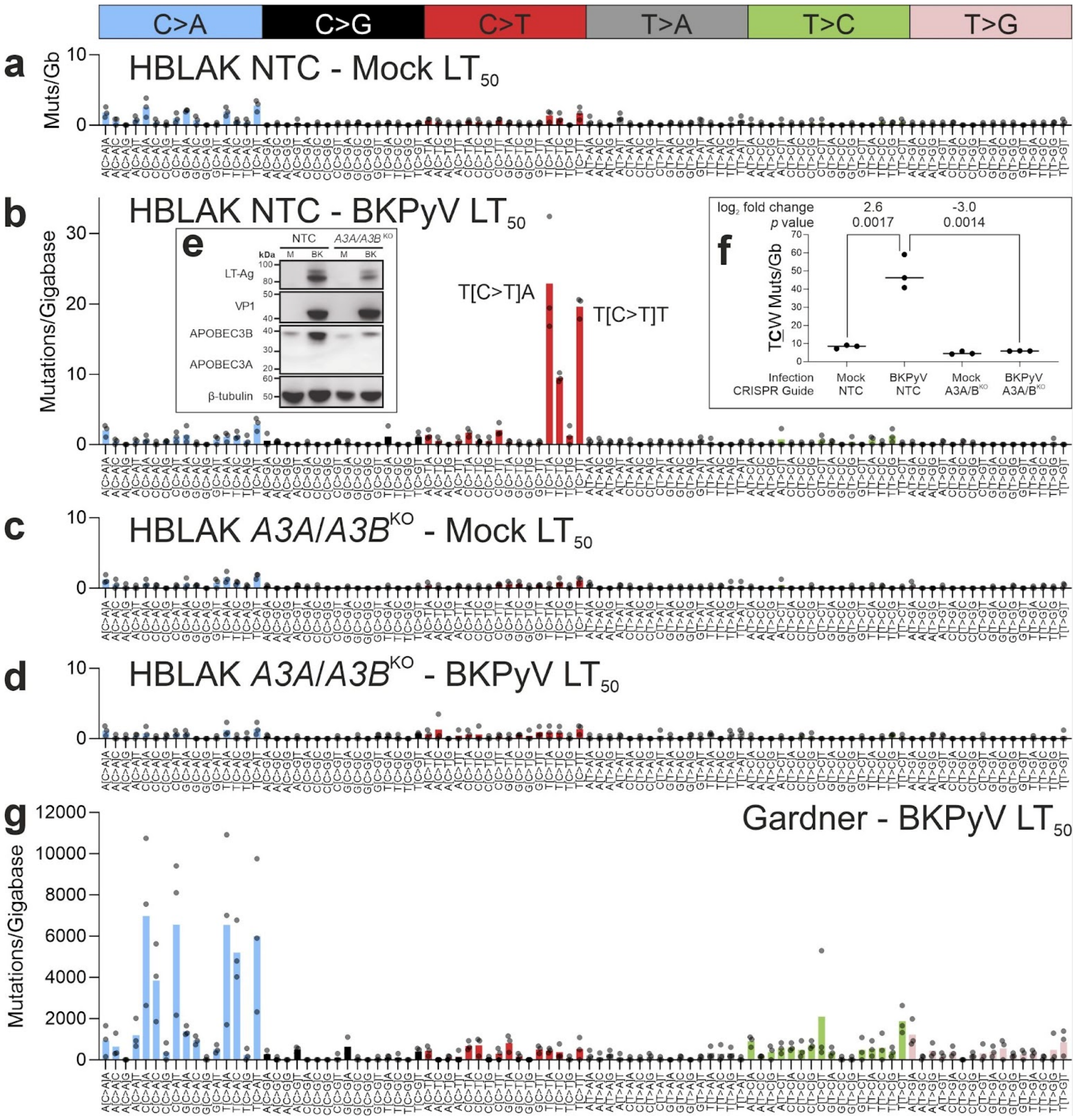

Fig. S8 - Full Single Base Substitution (SBS) mutational signatures derived from Nanorate sequencing of BKPyV (Gardner strain) MOI = 0.5 infection of the HBLAK urothelial cell line. All host HBLAK mutational signatures were collected at the lethal time 50 (“LT_{50}”, where 50% of the culture had succumbed to the cytopathic effects of infection) and are expressed as the count for each mutation type divided by the number of gigabases from the NanoSeq library that was sequenced (Muts/Gb). n=3 for each panel with bars denoting the mean and error bars showing the standard deviation. **a** Mock infection of a non-targeted control (NTC) cells. **b** BKPyV infection of NTC cells. **c** Mock infection of *APOBEC3A* and *APOBEC3B* knockout “*A3A/A3B*^{KO}” cells. **d** BKPyV infection of *A3A/A3B*^{KO} cells. **e** Exemplar western blot highlighting the induction of APOBEC3B and the absence of APOBEC3A during infection of NTC HBLAK cells. **f** Statistical comparisons by unpaired t test of APOBEC3A/B signature T<u>C</u>W mutations per gigabase (highlighted by labels in panel **b**). **g** Mutational signature observed in the Gardner strain BKPyV genome during infection of non-targeted control HBLAK cells (the matched host HBLAK genome signature is shown in panel **b**).

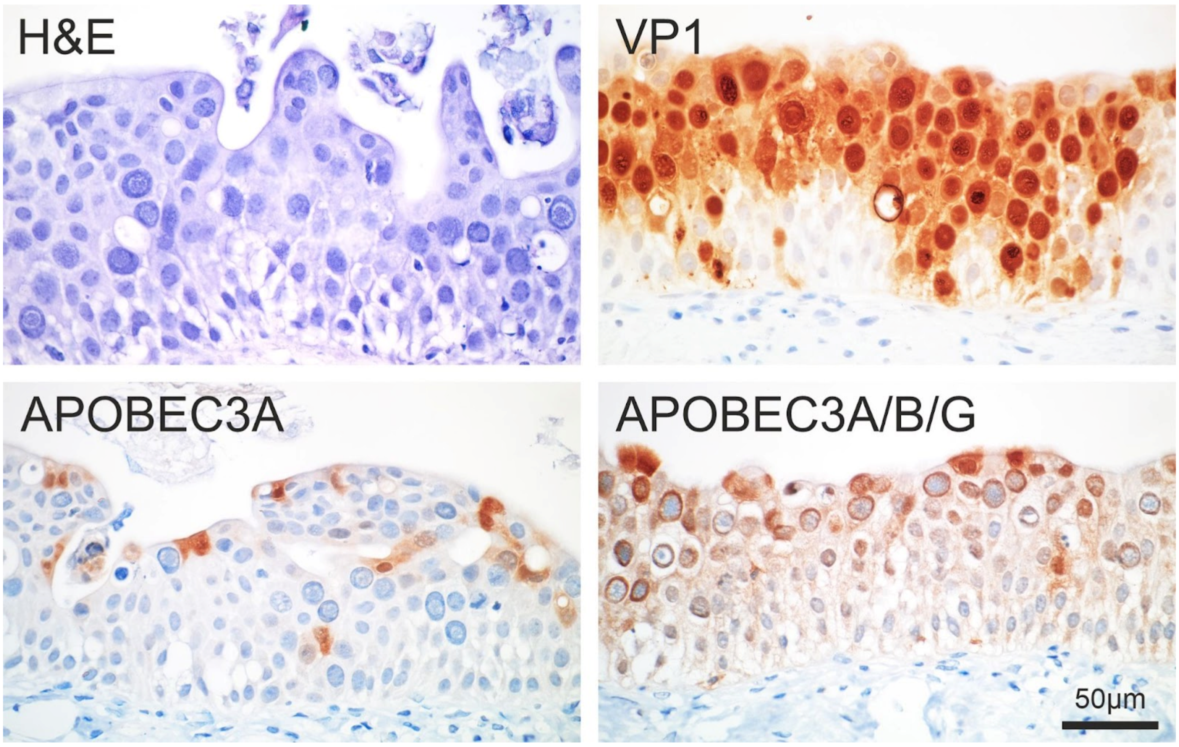

Fig. S9 – Haematoxylin and Eosin ("H&E") stained chronic BKPyV (Dunlop strain) infections of an organ culture model of normal human ureter show evidence of anisokaryosis and the nuclear inclusions first described by Sylvia Gardner and Anne Field using transmission electron microscopy (33). Immunoperoxidase labelling of the viral major capsid protein 1 (VP1) confirmed the nuclear inclusion bodies visualised by H&E were dense accumulations of virions. An APOBEC3A-specific antibody (18) rarely labelled cells containing nuclear inclusions but frequently labelled adjacent "bystander" cells. A monoclonal antibody that recognises a shared epitope in APOBEC3A/B/G (37) labelled both cells containing nuclear inclusions and adjacent "bystander" cells. Inside nuclei bearing nuclear inclusions the APOBEC3A/B/G-labelling was pushed to the peripheral regions of the nucleus. This exclusion of APOBECs from virion dense regions may offer additional protection to the viral progeny genomes. Based on the lack of induction of APOBEC3G during infections (fig. S2), we anticipate that APOBEC3B protein is responsible for the labelling within nuclei bearing nuclear inclusion bodies. Scale bar in the bottom right corner applies to all images.

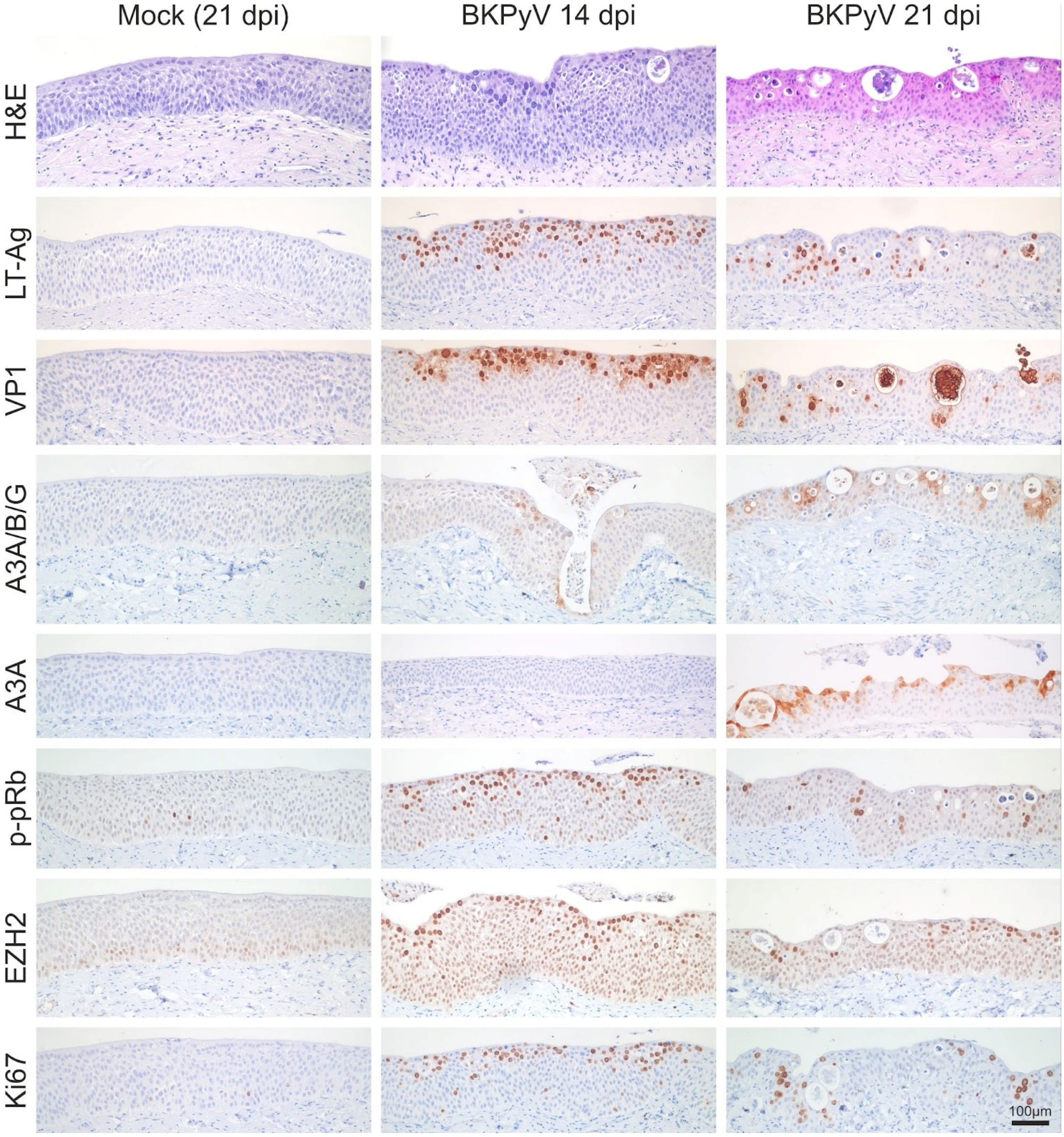

Fig. S10 – Histology and immunolabelling of normal human ureteric organ cultures infected apically with BKPyV (Dunlop) and assessed at 14 and 21 days post infection (dpi). Scale bar in bottom right applies to all images and this figure includes non-infected controls associated with **Fig. 4**. Haematoxylin and Eosin (H&E) staining highlights the larger, darker nuclei of infected cells that have been through S-phase. Large T antigen (LT-Ag) is the main effector protein of the *polyomaviridae* and immunoperoxidase labelling showed widespread nuclear labelling at 14 dpi. Viral major capsid protein 1 (VP1) labelling was widespread at 14 dpi and highlighted the encapsulation and apical extrusion of late-stage infection cells by the urothelium close to the lytic phase. Immunoperoxidase labelling with a monoclonal antibody that recognises a shared epitope in APOBEC3A/B/G (37) showed no detectable labelling at 14 dpi except around a single area of

apical extrusion. By 21 dpi APOBEC3-labelling was widespread surrounding areas of encapsulation and extrusion of infected cells.

Antibodies were used to label proteins associated with re-entry into the cell cycle by normally G0-arrested human urothelial cells (specific targets were phosphorylated$^{serine807/811}$- retinoblastoma protein "p-pRb", the Enhancer of zeste homolog 2 "EZH2" transcription factor and the cell cycle stage marker Ki67). We previously reported in dNHU cultures that these proteins are mainly detected during the early stages of BKPyV infection; however, some bystander cells also became positive (22).

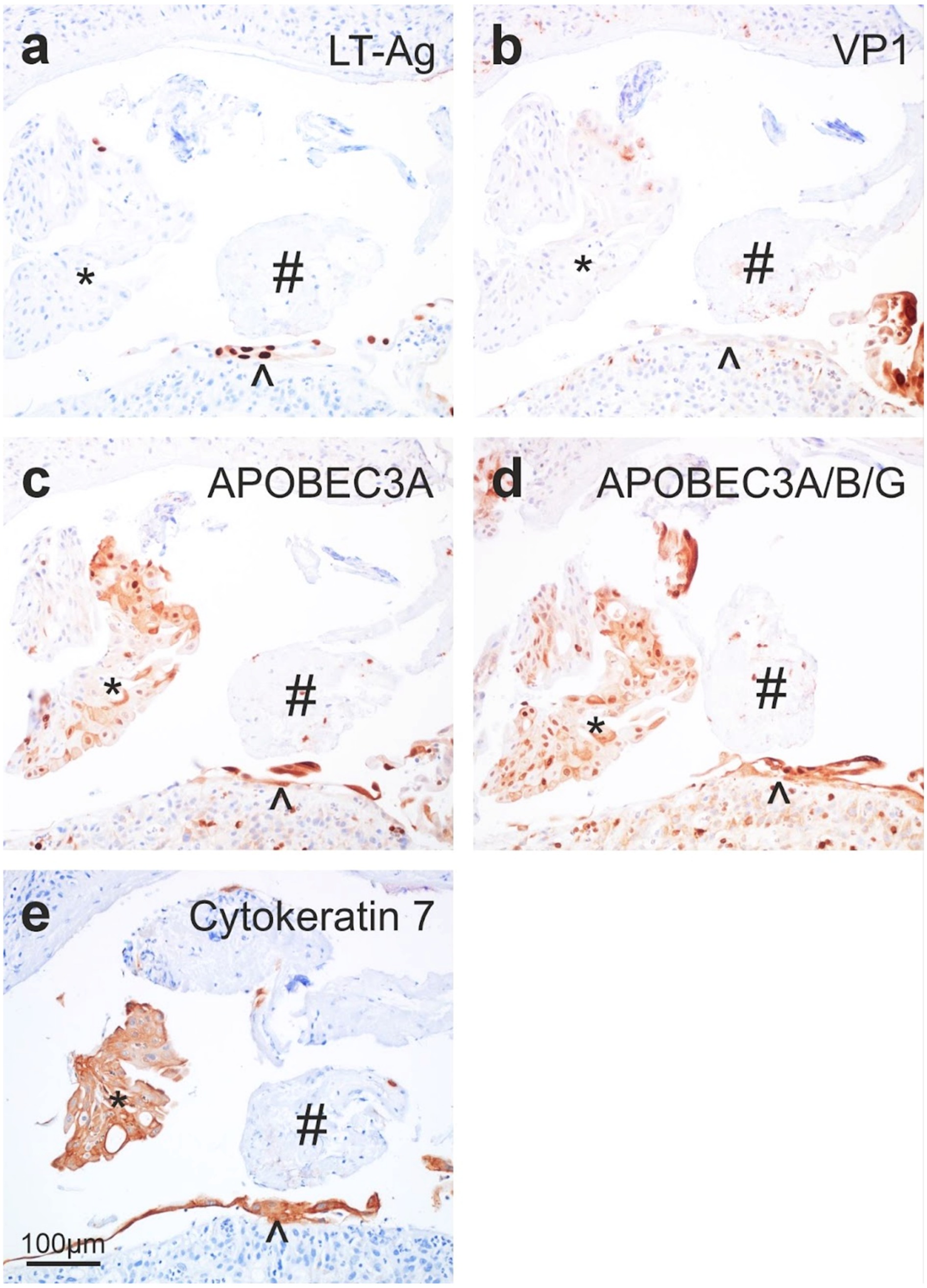

Fig. S11 – Histology of rare bladder biopsy from a bone marrow transplant recipient suffering from BKPyV-associated haemorrhagic cystitis. The biopsy contains some partially detached urothelium (denoted with *) despite widespread ulceration. Immunoperoxidase labelling is shown for **a** viral large T antigen (LT-Ag), **b** Viral major capsid protein 1 (VP1), **c** APOBEC3A monospecific, **d** APOBEC3A/B/G and **e** cytokeratin 7 (to highlight the urothelial cells). On serial sections, a proximal piece of stroma is denoted with a "#" and urothelium clearly attached to the basement membrane is show with the "^" symbol to provide spatial reference points. The urothelial cluster denoted* highlights positivity for APOBEC3 enzymes in cells that were negative for viral proteins. Scale bar shown in panel **e** applies to all images in this figure.

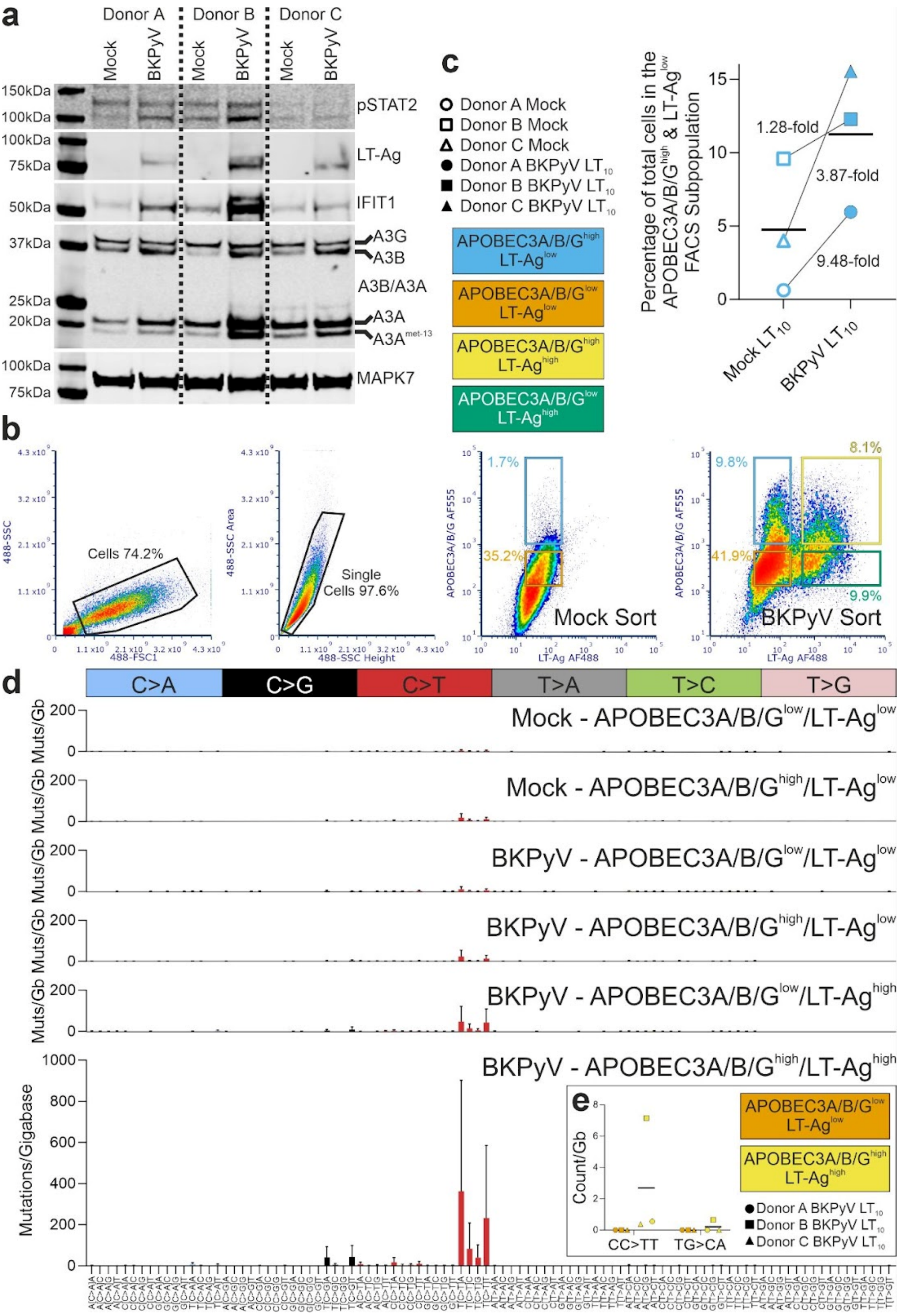
a
Donor A
Donor B
Donor C
Mock
BKPyV
150kDa
100kDa
75kDa
50kDa
37kDa
25kDa
20kDa
pSTAT2
LT-Ag
IFIT1
A3G
A3B
A3B/A3A
A3A
A3A met-13
MAPK7
c
Donor A Mock
Donor B Mock
Donor C Mock
Donor A BKPyV LT10
Donor B BKPyV LT10
Donor C BKPyV LT10
APOBEC3A/B/G high LT-Ag low
APOBEC3A/B/G low LT-Ag low
APOBEC3A/B/G high LT-Ag high
APOBEC3A/B/G low LT-Ag high
Percentage of total cells in the APOBEC3A/B/G high & LT-Ag low FACS Subpopulation
1.28-fold
3.87-fold
9.48-fold
Mock LT10
BKPyV LT10
b
488-SSC
488-FSC1
Cells 74.2%
488-SSC Area
488-SSC Height
Single Cells 97.6%
APOBEC3A/B/G AF555
LT-Ag AF488
1.7%
35.2%
Mock Sort
9.8%
8.1%
41.9%
9.9%
BKPyV Sort
d
C>A
C>G
C>T
T>A
T>C
T>G
Muts/Gb
Mock - APOBEC3A/B/G low/LT-Ag low
Mock - APOBEC3A/B/G high/LT-Ag low
BKPyV - APOBEC3A/B/G low/LT-Ag low
BKPyV - APOBEC3A/B/G high/LT-Ag low
BKPyV - APOBEC3A/B/G low/LT-Ag high
BKPyV - APOBEC3A/B/G high/LT-Ag high
Mutations/Gigabase
e
Count/Gb
CC>TT
TG>CA
APOBEC3A/B/G low LT-Ag low
APOBEC3A/B/G high LT-Ag high
Donor A BKPyV LT10
Donor B BKPyV LT10
Donor C BKPyV LT10

Fig. S12 - **a** Western blot analysis of lysates collected at 21 days post infection (dpi) with BKPyV (Dunlop strain) representing the three donor cell lines selected for FACS-NanoSeq at a timepoint where 10% of the infected culture has been lost to cytopathic effects LT_{10} (**Fig. 5**). Targets include Large T antigen (LT-Ag), a phosphorylated form of Signal Transducer and Activator of Transcription 2 (pSTAT2), APOBEC3 enzymes, interferon-induced protein with tetratricopeptide repeats 1 (IFIT1) and the house-keeping protein mitogen-activated protein kinase 7 (MAPK7). **b** Exemplar FACS gating of Donor A cells at LT_{10}. Initially gating was used to select cells (by FCC1/SSC dot plot using the 488 laser; far left) and single cells (by SSC-Height/SSC-Area dot plot using the 488 laser; centre left), prior to sorting for NanoSeq using the gates shown on APOBEC3A/B/G AF555 and LT-Ag AF488 for mock-infected dNHU cultures (centre right) and BKPyV-infected dNHU cultures (far right), respectively. The colour codes above the "Mock Sort" (centre right) panel apply to both mock- and BKPyV-infection sort gates and percentages. **c** The percentage of cells sorted by FACS into the APOBEC3A/B/G^{high}/LT-Ag^{low} subpopulation increased in all three donors (mean = 4.9-fold) when comparing mock to BKPyV infections (n=3). Bar denotes the mean and the fold change for each donor is given on the graph. **d** Full Single Base Substitution (SBS) mutational signatures derived from FACS-NanoSeq of subpopulations based on labelling for APOBEC3A/B/G (using the 5210-87-13 antibody (37)) and LT-Ag (using the Pab108 antibody). **e** Double Base Substitution (DBS) analysis focussed on FACS-NanoSeq of BKPyV (Dunlop) infected culture subpopulations labelled APOBEC3A/B/G^{low} LT-Ag^{low} (orange) in comparison with APOBEC3A/B/G^{high} LT-Ag^{high} (yellow) hints at the emergence of a signature with CC>TT and TG>CA changes suggestive of COSMIC signature "DBS11".

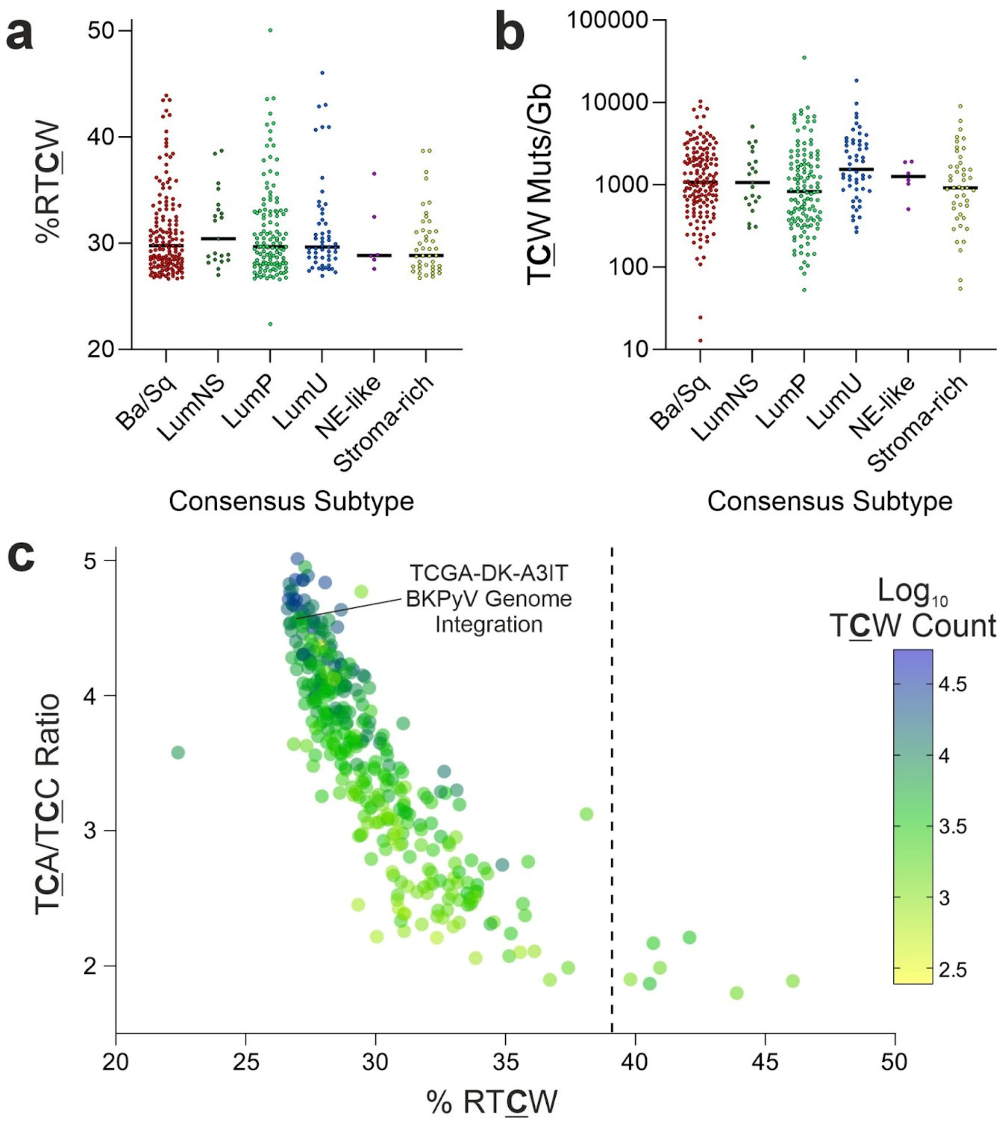

Fig. S13 - Whole genome sequencing data for The Cancer Genome Atlas (TCGA) muscle invasive bladder cancer (MIBC) cohort n=409 (21), showing the molecular subtypes according to the current consensus classification (81). **a** No significant difference in the %RTCW mutations was observed between the consensus molecular subtypes of MIBC suggesting a shared mechanism of mixed APOBEC3A and APOBEC3B mediated carcinogenesis, with the former dominating the process (n=402 (21, 81)). **b** No significant difference in TCW APOBEC signature mutational load between the MIBC consensus molecular subtypes (n=402 (21, 81)). **c** Tumours with the highest similarity to the pure APOBEC3A over-expression Hap1 models (18) (**Fig. 6**; high TCA/TCC ratio and low % RTCW) had the highest number of TCW APOBEC signature mutations. This analysis retained only samples with >16.8% of mutations derived from TCW (n=348). One sample "TCGA-DK-A3IT" had the BKPyV genome integrated into that of the host and is highlighted on the plot as having a robust APOBEC3A-like pattern of mutations.

Antigen	Clone/ID	Host	Supplier	Catalogue Number	Lot Number	Western Blot Dilution	FACS Dilution	Immunofluorescence Dilution	Immunoperoxidase Dilution	Formalin Antigen Retrieval
APOBEC3A	UMN13	Rabbit	Gift from Reuben Harris	N/A	N/A			1:400	1:1000	Citric Acid Buffer pH6
APOBEC3A, APOBEC3B, APOBEC3G	5210-87-13	Rabbit	Gift from Reuben Harris	N/A	N/A	1:800	1:50	1:100	1:400-1:2000	Citric Acid Buffer pH6
Cytokeratin 7	OVTL	Mouse	Hycult Biotech	HM2128	23382M1117-A				1:400	Citric Acid Buffer pH6
EZH2	D2C9	Rabbit	Cell Signaling	5246	7				1:50	Citric Acid Buffer pH6
Goat-anti-Mouse Alexa488	N/A	Goat	Invitrogen	A11001	2015565		1:250	1:250		
Goat-anti-Rabbit Alexa555	N/A	Goat	Invitrogen	A21428	1937183		1:1000	1:1000		
IFIT1	OTI3G8	Mouse	Abcam	ab118062	GR3245141-24	1:1000		1:100		
Ki67	MM1	Mouse	Leica	NCL-L-Ki67-MM1	6091582				1:600	Citric Acid Buffer pH6
Large T Antigen (LT-Ag)	Pab 108	Mouse	Santa Cruz	sc-148	N/A	1:250	1:100	1:200	1:200	Citric Acid Buffer pH6
Retinoblastoma (phosphorylated Serine 807/811)	N/A	Rabbit	Cell Signaling	9308	6				1:500	Citric Acid Buffer pH6
STAT1 (phosphorylated)	58d6	Rabbit	Cell Signaling	9167	18	1:1000				
STAT2 (phosphorylated)	D3P2P	Rabbit	Cell Signaling	88410	5	1:1000		1:50		
Total ERK (MAPK7 analysed)	pan-ERK clone 16	Mouse	Becton-Dickinson	610123	1060797	1:2000				
Viral Protein 1 (VP1)	Pab 597	Mouse	Gift from Chris Buck	N/A	N/A	1:250		1:200	1:200	Citric Acid Buffer pH6

Table S1.

Details of the antibodies used in this study.
